# Alloyed Pt_3_M (M = Co, Ni) nanoparticles supported on S- and N-doped carbon nanotubes for the oxygen reduction reaction

**DOI:** 10.3762/bjnano.10.125

**Published:** 2019-06-21

**Authors:** Stéphane Louisia, Yohann R J Thomas, Pierre Lecante, Marie Heitzmann, M Rosa Axet, Pierre-André Jacques, Philippe Serp

**Affiliations:** 1 LCC-CNRS, Université de Toulouse, CNRS, INPT, Toulouse, France; 2 Université Grenoble Alpes, CEA-LITEN/DEHT, 17 rue des martyrs 38000 Grenoble, France; 3 CEMES-CNRS, 29 rue Jeanne Marvig, 31055 Toulouse Cedex 4, France

**Keywords:** carbon nanotubes, cobalt, ionic liquid, nickel, oxygen reduction reaction, platinum, proton exchange membrane fuel cell (PEMFC)

## Abstract

Sulfur- (S-CNT) and nitrogen-doped (N-CNT) carbon nanotubes have been produced by catalytic chemical vapor deposition (c-CVD) and were subject to an annealing treatment. These CNTs were used as supports for small (≈2 nm) Pt_3_M (M = Co or Ni) alloyed nanoparticles that have a very homogeneous size distribution (in spite of the high metal loading of ≈40 wt % Pt), using an ionic liquid as a stabilizer. The electrochemical surface area, the activity for the oxygen reduction reaction and the amount of H_2_O_2_ generated during the oxygen reduction reaction (ORR) have been evaluated in a rotating ring disk electrode experiment. The Pt_3_M/N-CNT catalysts revealed excellent electrochemical properties compared to a commercial Pt_3_Co/Vulcan XC-72 catalyst. The nature of the carbon support plays a key role in determining the properties of the metal nanoparticles, on the preparation of the catalytic layer, and on the electrocatalytic performance in the ORR. On N-CNT supports, the specific activity followed the expected order Pt_3_Co > Pt_3_Ni, whereas on the annealed N-CNT support, the order was reversed.

## Introduction

Proton exchange membrane fuel cells (PEMFCs) convert chemical energy from the hydrogen oxidation reaction (HOR) and the oxygen reduction reaction (ORR) into electrical energy. PEMFCs are one of the most promising technologies in the field of renewable energy (and especially for transport applications), but the cost and lifetime are factors still to be improved in order to achieve widespread dissemination of this technology [1–2]. Due to the sluggish reaction kinetics for the ORR, the cathode active layer contains generally four times more catalyst than the anodic layer, which explains why most of the research is focused on the optimization of the cathodic catalytic layer. Platinum nanoparticles (NPs) supported on carbon black (CB), especially Vulcan XC-72 [3–4], are usually used as the catalyst. To meet the performance and durability requirements for transport applications, a metal loading of 0.4 mg_Pt_·cm^−2^ for the cathode side is commonly used, which explains the high cost of these systems [5]. One lever to reduce the cost of this technology is the reduction of the cathode Pt loading, but this must be done without compromise to the catalyst layer performance and durability.

It is known that catalyst degradation via platinum dissolution and carbon corrosion plays an important role in the voltage degradation of PEMFCs [6–8]. CB, which is widely used, particularly for its low cost, suffers from thermochemical instability and corrosion in fuel cell applications. In the cathodic layer, the oxidizing, wet and acidic environment, the high electrochemical potential, and the high platinum loading all lead to the oxidation of the carbon surface, and occasionally to the formation of CO_2_ [9]. This carbon corrosion modifies the mass transport properties of the active layer, especially for the water management, and accelerates the degradation of the Pt NPs [10–11].

One way to reduce the Pt content is to use more active, tailored NPs [12–13], for example, bimetallic NPs with a core–shell structure [14–15]: a Pt shell can be deposited on a low-cost transition metal such as Co [16–18], Ni [19–20] or Cu [21] or their nitrides [22]. Kristian et al. have described a redox–transmetalation method for the synthesis of Co_core_–Pt_shell_ particles with a high activity for the ORR [23]. Platinum-based alloys can also been used [24–25]. Therefore, it seems important to develop nanostructured catalysts supported on a material with electronic conductivity and surface area close to the common CB but with more resistance towards corrosion. Interestingly, it has recently been shown that the introduction of small amounts of ionic liquids (ILs), which are known as NP stabilizers, including on carbon supports [26], into Pt-based catalysts can further improve the ORR performance. This is likely due to the high O_2_ solubility in the IL phase [27]. It was also demonstrated that the choice of the carbon support, in combination with ILs, is also important to achieve high Pt dispersion, and functionalized carbons should be preferred, presumably because of their stronger interaction with the IL [28].

Carbon nanotubes (CNTs) are well known for their remarkable chemical and physical properties and appear to be an interesting alternative to replace CB in fuel cell applications [29–30]. It has been described that CNTs could be used as resistant material to support nanostructured PtNi hollow particles, but it appears that the structure of the used CNT might be responsible for the large external diameter of the deposited particles, which is close to 25 nm [31]. Previous works have shown the possibility to dope CNTs with nitrogen (N-CNT) or sulfur (S-CNT) in order to modify properties such as electronic conduction and surface chemistry [32–34]. This strategy contributes to improve the metal dispersion and to increase the performance of the catalyst for the ORR due to the structural and electronic properties of the doped CNT [34–35]. Additionally, the amount of heteroatom in the doped structure has an effect on the hydrophobicity of the material, which could provide a solution to facilitate the water management in the active layer. In fact, water management in the cathodic layer is a key challenge: the electrolytic membrane has to be hydrated enough to favor the proton conduction, but an excess of water in the cathodic layer will decrease the oxygen accessibility to the active sites. Thermal treatment can be used to improve the carbon corrosion resistance of the CNT. In fact, annealing at high temperature (above 1000 °C) is used to remove structural defects from the CNT in order to obtained more stable [36] and more conductive [37] structures. In the case of the N-CNTs, the thermal annealing can also modify the ratio of the different nitrogen groups at the surface [38], and consequently, the metal–support interaction.

This work proposes: i) to reduce the amount of Pt on the catalyst by using Pt alloyed compounds, and ii) to increase the support corrosion resistance using heat-treated carbon materials. Several CNT materials have been synthetized, fully characterized (structural and surface properties) and used as catalyst supports. CNT-supported bimetallic Pt_3_Co or Pt_3_Ni NPs were synthetized. To evaluate catalyst performance, electrochemical characterization was performed using a rotating ring disk electrode (RRDE) experiment to determine the active surface and the activity for the ORR of each catalyst. Finally, membrane electrode assemblies (MEAs) of 25 cm^2^ active area, integrating the synthesized catalysts, have been prepared and tested. The one giving the best beginning-of-life performance has been aged following a recommended accelerated stress test (AST) cycle for catalyst support corrosion.

## Results and Discussion

### Synthesis and characterization of the CNTs and Pt_3_M/CNT

Three kinds of CNTs have been produced by catalytic chemical vapor deposition (c-CVD): undoped (CNTs), N-doped (N-CNTs) and S-doped CNT (S-CNTs). To further increase the corrosion resistance and the electrical conductivity of the N-CNTs, they were annealed at 1000 °C to produce N-CNT_HT_.

**Figure 1:**
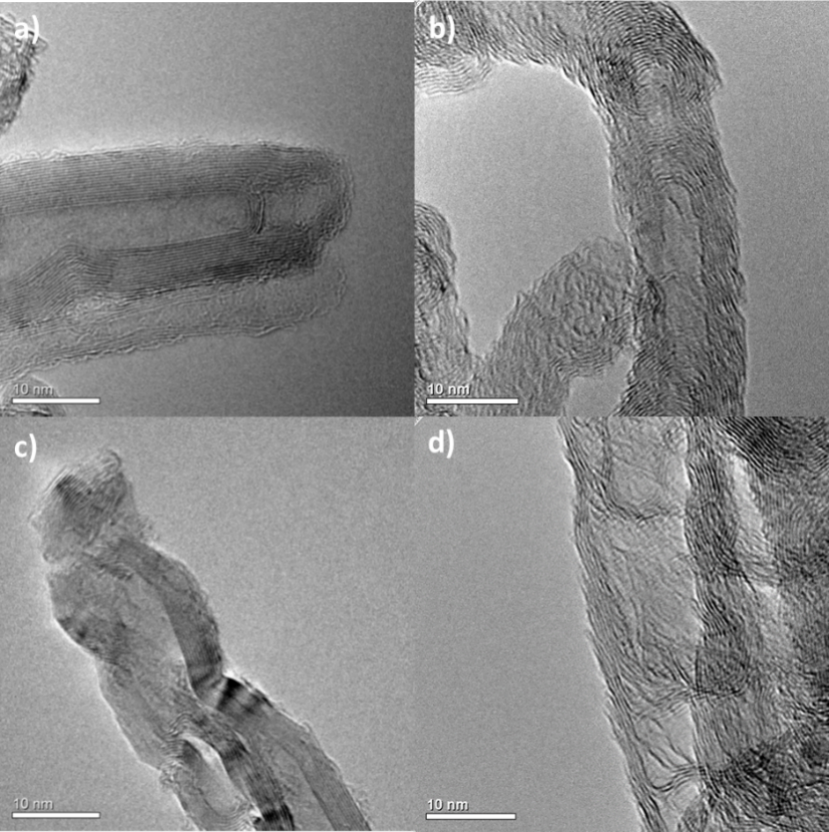
HRTEM micrographs of: a) CNTs; b) N-CNTs; c) S-CNTs, and d) N-CNTs_HT_. (Scale bar = 10 nm).

The introduction of nitrogen or sulfur into the CNT structure has an effect on the structural properties of the prepared materials. High resolution transmission electron microscopy (HRTEM) analysis shows a remarkable difference between the carbon structures synthetized (Figure 1).

Very regular structures were obtained for the CNT sample (Figure 1a), while the N-CNT sample presented a “bamboo-like” structure typically found in N-doped CNTs (Figure 1b) [39], and the structure of the S-doped CNTs presents some alterations (bulbous segments, Figure 1c), which are different than those observed for the N-CNTs (bamboo structure). The N-CNTs_HT_ show similar structure to the HRTEM observations (Figure 1d). Low magnification TEM micrographs of the carbon supports are given in Supporting Information File 1, Figure S1. The CNTs, N-CNTs and S-CNTs have mean external diameters of 15 ± 5, 18 ± 8, and 15 ± 7 nm, respectively. The specific surface area (SSA) of these materials ranged between 150 m^2^/g (CNTs) and 190 m^2^/g (S-CNTs).

Structural characterization was performed using Raman spectroscopy and X-ray diffraction (XRD, see Table 1 and Supporting Information File 1, Figure S2 for Raman spectra). In Raman spectroscopy, a useful parameter for carbon nanotubes is the ratio between the D band (*I*_D_) at ≈1380 cm^−1^, attributed to the defects of the CNT structure, and the G band (*I*_G_) at ≈1580 cm^−1^, the first-order Raman band of all sp^2^ carbon materials. The presence of disorder in CNTs can also impact: i) the intensity of other bands, such as the G’ band at ≈2700 cm^−1^, and ii) the position and shape of the peaks [40]. The G’ band is indicative of long-range order in a sample. Finally, another parameter, measurable by Raman spectroscopy that is relevant to catalyst preparation, is the L_D_: L_D_ is a typical inter-defect distance that we have measured as described in [41]. A lower *I*_D_/*I*_G_ (and higher L_D_) is obtained for the CNT sample and a higher *I*_D_/*I*_G_ (and lower L_D_) for the N-CNT sample, which is in accordance with the TEM observations. The N-CNT_HT_ sample shows a decrease in the number of defects compared to N-CNT, as expected after the high temperature treatment. S-CNTs constitute an intermediate situation. The d_002_ inter-planar spacing results obtained with XRD are presented in Table 1.

**Table 1:** Chemical, textural, and crystalline properties of the carbon supports.

Supports	XPS analysis				Textural properties		Elemental analysis		
			
	C (%)	O (%)	N (%)	S (%)	BET surface area (m^2^·g^−1^)	Pore volume (cm^3^·g^−1^)	C (%)	N (%)	S (%)

CNT	98.4	1.6	–	–	151	2.6	92.3	–	–
N-CNT	91.2	4.9	3.9	0.2	182	2.7	92.3	2.9	–
N-CNT_HT_	94.1	3.5	2.4	–	168	2.4	95.6	1.6	–
S-CNT	95.6	2.8	–	1.3	190	1.1	78.6	–	5.0

	Raman analysis				Crystallite properties				
					
	*I*_D_/*I*_G_	*I*_D_/*I*_G_	*I*_D_/*I*_G_	*I*_D_/*I*_G_	d_002_ (nm)	d_002_ (nm)			

CNT	0.87	0.87	0.87	0.87	0.3444	0.3444			
N-CNT	1.02	1.02	1.02	1.02	0.3436	0.3436			
N-CNT_HT_	0.95	0.95	0.95	0.95	0.3379	0.3379			
S-CNT	0.99	0.99	0.99	0.99	0.3422	0.3422			

All the values are larger than that of graphite (3.334 Å), and the smallest value is obtained for N-CNT_HT_, indicating a higher level of graphitization for this sample.

The elemental composition as well as the surface chemistry is also affected by heteroatom doping. Elemental and X-ray photoelectron spectroscopy (XPS) analysis results are shown in Table 1. The elemental analysis confirmed the efficiency of the doping, and showed that S-CNT contains a significant amount of residual catalyst (iron, encapsulated in the structure of the tubes). An effect of heat treatment on the N-CNTs (besides the reduction of disorder) is to decrease the amount of nitrogen, which decreases from 2.9 to 1.6%. XPS analysis confirmed the bulk analyses and showed that the S-CNT sample also contains a significant amount of surface oxygen groups. The introduction of oxygen may correspond to the oxidation of the sulfur species introduced during the doping during the purification step in H_2_SO_4_. As the S 2p peaks are typically presented in spin–orbit doublets of S 2p_3/2_ and S 2p_1/2_ (splitting magnitude ≈1.18 eV), four S 2p_3/2_ peaks representing sulfur bonding of FeS_2_ (≈162.5 eV), H–S–C (≈163.5 eV), R–S–C (≈164.5 eV), and S–O (≈168.0 eV) were observed (Supporting Information File 1, Figure S3) [42–43]. The intense peak at 163.5 eV indicated the doping of CNTs with mainly thiol surface groups (Table 2). The peak at 164.5 eV could arise from the presence of sulfur in the carbon matrix, while the oxidation of surface thiols should produce S–O bonds (peak at 168 eV). The presence of pyrite could arise from the significant amount of remaining iron catalyst in this sample. Different nitrogen groups are present in N-CNT, and the proportion of these groups was determined by deconvolution of the main N 1s peak: pyridinic nitrogen, pyrrolic nitrogen, quaternary nitrogen and nitrogen-oxidized species were identified (Table 2, and Supporting Information File 1, Figure S4). XPS analysis also showed the presence of sulfonic acid groups on the surface of N-doped CNTs (Supporting Information File 1, Figure S4b), and the S content increases with the nitrogen content. This functionalization occurs during the purification step with sulfuric acid. In the N-CNT_HT_ sample, we measured a clear decrease of the nitrogen-oxidized species.

**Table 2:** Contribution of species detected by deconvolution of XPS spectra for several N-CNT samples.

Sample	Surface groups			

S-doped	S_FeS2_ (%)	S_thiol_ (%)	S_sulphide/thioether_ (%)	S_ox_ (%)

S-CNT	5.0	64.9	22.8	7.3

N-doped	N_pyridinic_ (%)	N_quaternary_ (%)	N_pyrrolic_ (%)	N_oxidized_ (%)

N-CNT	23.9	23.4	9.1	43.6
N-CNT_HT_	30.9	54.3	10.3	4.5

Electrocatalyst support materials are crucial to both the performance and durability of PEM fuel cells [44–45]. These materials should combine some key characteristics such as: i) an adapted surface chemistry to allow high dispersion of the metallic phase at very high metal loading, ii) a good balance between hydrophobicity and hydrophilicity to allow water management and interaction with the electrolyte, iii) a good dispersibility in the ink to limit mass transfer, and iv) structural features allowing high conductivity and chemical stability. As some of these characteristics are not compatible (e.g., a high metal dispersion should be favored on defective carbon supports but this should be detrimental to the stability and electronic conduction), some compromises have to be made.

**Figure 2:**
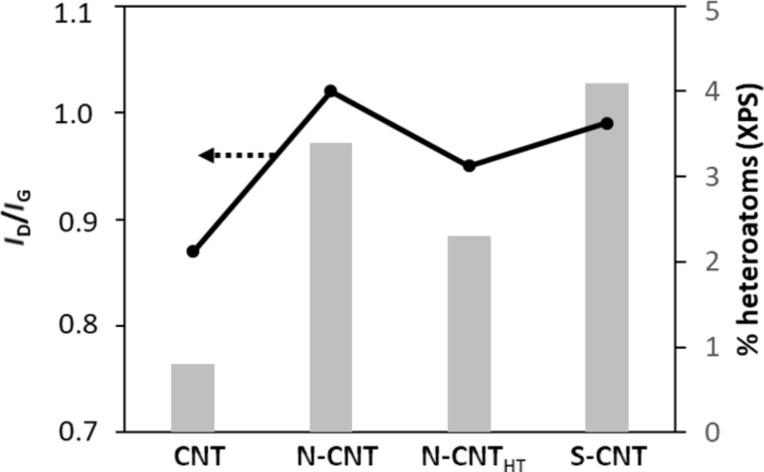
Evolution of the *I*_D_/*I*_G_ ratio (from Raman spectroscopy) and the percent of surface heteroatoms (from XPS) in the investigated CNT supports.

Figure 2 shows the evolution of the *I*_D_/*I*_G_ ratio from Raman spectroscopy and the percent of surface heteroatoms from XPS in the investigated supports. The *I*_D_/*I*_G_ ratio reflects the concentration of defects in these supports, where a high ratio favors metal dispersion. A high percentage of surface heteroatoms should favor metal dispersion and interaction with the electrolyte but may have negative impacts on the electronic conductivity, the stability, and can modify the metal/support interactions and thus the electrocatalytic performance. From this figure, we can see that the CNTs and S-CNTs constitute the extremes and that the compromise could lie in the N-doped nanotubes, particularly the N-CNT_HT_.

**Figure 3:**
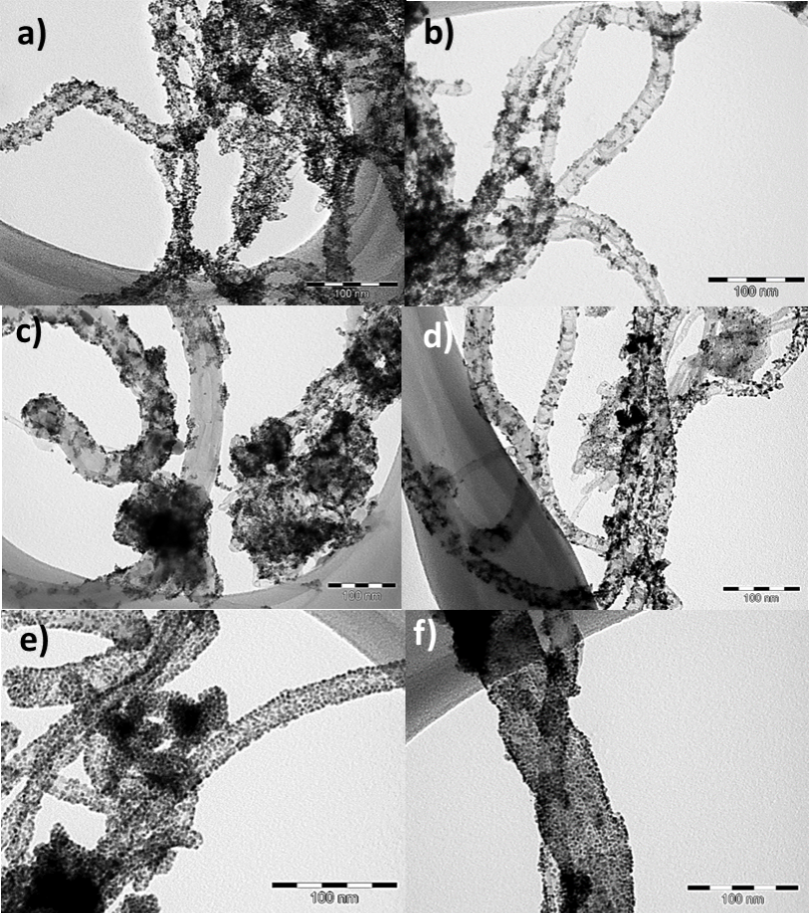
TEM micrographs of: a) Pt_3_Co/N-CNT; b) Pt_3_Co/N-CNT_HT_; c) Pt_3_Co/S-CNT; d) Pt_3_Ni/N-CNT; e) Pt_3_Ni/N-CNT_HT_, and f) Pt_3_Ni/S-CNT. Scale bar = 100 nm.

These CNTs have been used to support Pt_3_M (M = Co, Ni) NPs. The bimetallic NPs were prepared using the transmetalation method described by Kristian et al. [23]. The redox couple used for the transmetalation is Co^2+^(II)/Co(0) (*E*° = −0.77 V/RHE) and PtCl_4_^2−^/Pt(0) (*E*° = 0.67 V/RHE) for Pt_3_Co NPs. In our procedure, the ammonium salt used as a stabilizer has been replaced by an ionic liquid in order to obtain a better distribution of NPs at the surface of the CNTs [46]. Indeed, the use of hexadecyl trimetyl ammonium bromide leads to the formation of small NPs (1.90 ± 0.77 nm) that are not well-dispersed on the support (see Supporting Information File 1, Figure S5a for the Pt_3_Co/CNT sample). In order to improve the NP distribution on the support, [bmim][Tf_2_N] was chosen as stabilizer. It is known that imidazolium salts show good interaction with CNTs due to π–π interactions and could also be used as a stabilization agent for NPs [46–48]. The TEM images of sample Pt_3_Co/CNT (Supporting Information File 1, Figure S5b) show that the use of ILs does not result in well-dispersed NPs on this support. The presence of heteroatoms in the CNTs significantly affects the distribution of the NPs on the CNT surface (Figure 3a–c). N-doped or S-doped structures are known to be more reactive due to the presence of different functional groups at the surface of the CNTs, while un-doped CNTs are relatively inert. With nitrogen or sulfur groups acting as nucleation centers, the distribution of NPs on the CNT walls is better on N-CNTs and S-CNTs than on CNTs. A decrease of the nitrogen content was observed with annealing of the N-CNTs. This could explain why the NP distribution is better for Pt_3_Co/N-CNT compared to Pt_3_Co/N-CNT_HT_. Non-annealed CNTs seem to have better interaction with the Co precursor and the as-obtained catalysts show a better NP distribution. Nevertheless, the mean size of the NPs is around 2 nm for all the prepared catalysts (Table 3 and Supporting Information File 1, Figure S6).

**Table 3:** Mean nanoparticle size and metal loading for PtM/CNT catalysts.

Sample	Particle size (nm)	M (wt %)	Pt (wt %)

Pt_3_Co/CNT	2.28 ± 0.82	12.5	33.5
Pt_3_Co/N-CNT	1.71 ± 0.73	15.4	41.5
Pt_3_Co/N-CNT_HT_	2.08 ± 0.90	7.9	40.2
Pt_3_Co/S-CNT	2.00 ± 0.71	15.4	33.0
Pt_3_Ni/N-CNT	2.42 ± 1.24	15.3	40.3
Pt_3_Ni/N-CNT_HT_	1.87 ± 0.86	18.3	42.3
Pt_3_Ni/S-CNT	2.30 ± 0.80	14.8	23.3

Pt_3_Ni/N-CNT catalysts were prepared using the same procedure. The redox couple used for the transmetalation is Ni^2+^(II)/Ni(0) (*E*° = −0.257 V/RHE) and PtCl_4_^2−^/Pt(0) (*E*° = 0.67 V/RHE). A poor metal distribution was obtained on the CNTs, thus Pt_3_Ni NPs were further studied only on N-CNTs and S-CNTs. For the Pt_3_Ni catalysts, the mean size of the NPs is around 2 nm as observed for Pt_3_Co (Figure 3d–f and Supporting Information File 1, Figure S6). From these syntheses, we can estimate the influence of the metal precursor on the NP distribution. Regardless of the carbon support, much better distribution on the CNT walls was obtained with the Ni precursor. The affinity between the metal precursor and the carbon surface seems to play a role on the active phase distribution. The higher affinity of Ni compared to Co for graphene [49–50] and N-doped carbon [51–52] surfaces has already been reported in the literature and could hint at the origin of our results. The annealing of the N-CNTs has also an impact, since the Pt_3_Ni/N-CNT_HT_ catalyst shows a better distribution than the Pt_3_Ni/N-CNT one. We suspect that the annealing of the N-CNTs and the as-obtained modifications of the chemical surface causes an important change in the adsorption and diffusion of the metal. It has been shown that the binding energy between a transition metal and a carbon support depends on the nature of the metal but also on the carbon material composition [53].

Particularly, Kattel showed that the binding energy of several transition metals could change if they are bound to two carbon atoms (M–C_2_), one carbon atom and one nitrogen atom (M–CN) or two nitrogen atoms (M–N_2_) [52]. Good metal distribution was also obtained on the S-CNT sample; in this case, however, the Pt loading is rather low (≈23%) compared to the N-CNTs. A recent study has shown that good metal distribution can be obtained on S-CNTs in the case of Pt catalysts (Pt loading = 20%, Pt NP size = 2.4 nm) [32–33]. The mean particle size for the Pt_3_Ni series is also around 2 nm, highlighting the effectiveness of the synthetic procedure followed in this work.

Moreover, these binding energies are also dependent on the nature of the metal. These observations could explain the strong differences between Pt_3_Co and Pt_3_Ni catalysts. In both cases, the theoretical Pt loading (50 wt %) is never reached, while the Co (or Ni) loading is higher than expected (theoretical content ≈7 wt %), strongly suggesting the presence of residual Co (or Ni) in these catalysts.

### RRDE measurements of electrochemical surface area (ECSA) and ORR activity and selectivity

In order to proceed to a first screening of the as-prepared catalysts, their electrochemical properties were evaluated by RRDE measurements. It is worth noting that electrocatalyst investigations are usually performed with a rotating ring-disk electrode (RRDE) in acidic or alkaline media. Previous works have shown that the ORR activities of Pt catalysts are strongly dependent on the electrolyte [54]. According to these studies, activities were found to increase from H_2_SO_4_ to HClO_4_ due to the specific effect of the adsorbed anion on different Pt(*hkl*) sites. Furthermore, the thin film RRDE method, with a low Nafion amount, is recommended to avoid diffusion resistance into the deposited active layer [55]. Therefore, low catalyst loadings are known to give higher activities, but these conditions are not always representative of the true working of the PEMFC [56]. Furthermore, the difficulty of dispersing CNTs in a highly diluted ink without using a dispersing agent is a real issue, which is why we have preferred to use a higher catalyst loading (100 µg_Pt_·cm^−2^).

**Figure 4:**
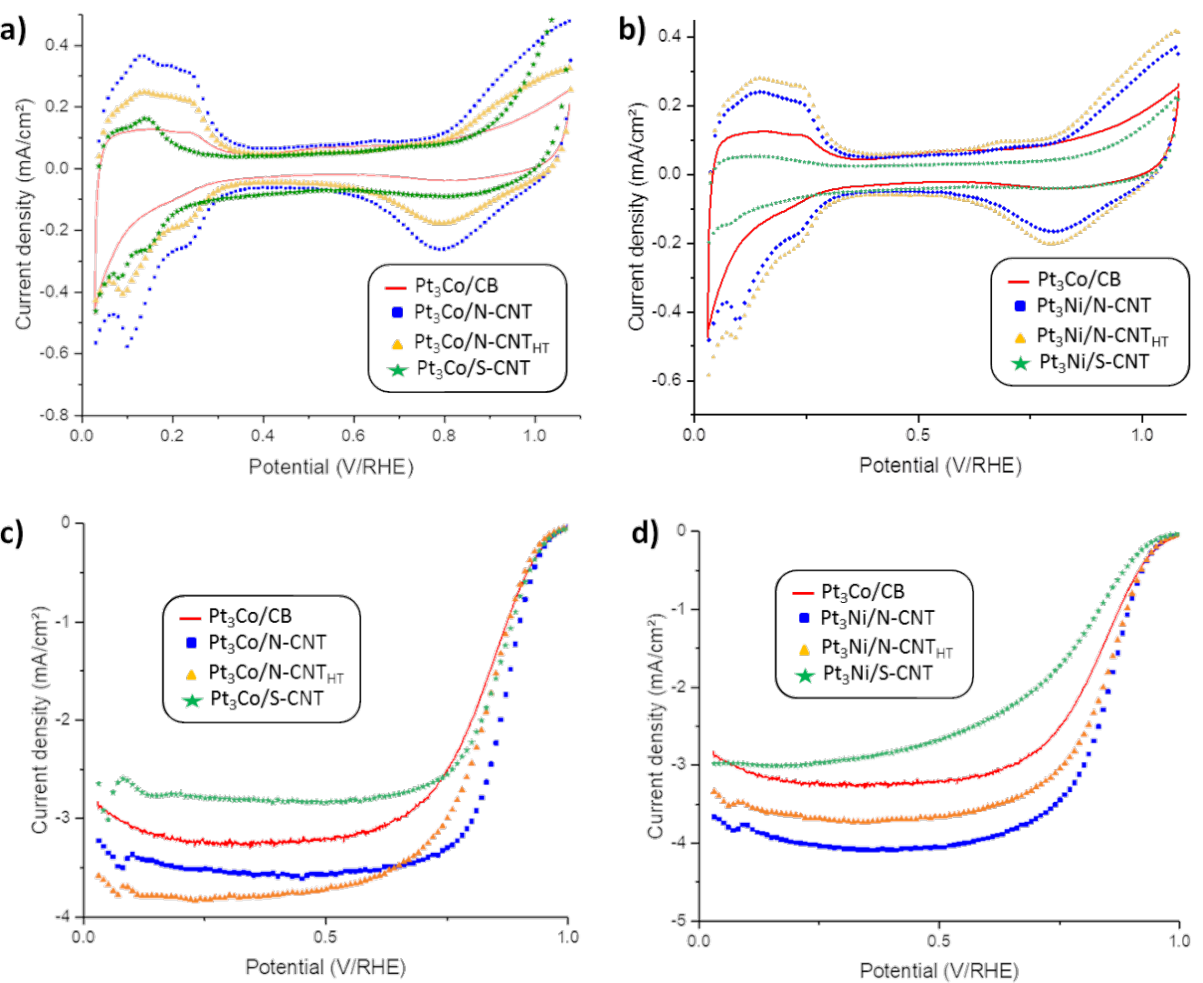
CVs from an RRDE experiment for the catalysts in 0.5 M H_2_SO_4_ at 5 mV·s^−1^ under N_2_ at 25 °C for a) the Pt_3_Co/CB and Pt_3_Co/CNT series and b) the Pt_3_Co/CB and Pt_3_Ni/CNT series. Background of linear sweep voltammetry (LSV) with RRDE experiment for the catalysts in 0.5 M H_2_SO_4_ at 5 mV·s^−1^ at 900 rpm under O_2_ at 25 °C for the c) Pt_3_Co/CNT and d) Pt_3_Ni/CNT series.

For Pt_3_Ni and Pt_3_Co catalysts, the typical specific activities (expressed as kinetic current densities) for ORR at 25 °C are in the range 2.5–4.5 mA·cm^−2^ in HClO_4_ [57–59] and around 0.3–0.7 mA·cm^−2^ in H_2_SO_4_ [59–61]. Interestingly, it was also reported that for Pt_3_Ni and Pt_3_Co catalysts in H_2_SO_4_, the activity increases in the order Pt_3_Ni > Pt_3_Co, and in HClO_4_ the order of activity at 25 °C was PtCo > PtNi [59]. However, these tendencies can also be particle size dependent. Thus, it was demonstrated that in H_2_SO_4_, this order (Pt_3_Ni > Pt_3_Co) is respected for particles >6 nm, whereas the opposite order prevails for particle sizes smaller than 6 nm [60]. In our work, the electrochemical properties were evaluated in H_2_SO_4_ and compared with a commercial Pt_3_Co/Vulcan XC-72 (Pt_3_Co/CB) catalyst. This commercial reference has shown excellent activity in RRDE and during single cell tests [5]. Figure 4a,b shows the cyclic voltammetry (CV) curves obtained in N_2_-purged 0.5 M H_2_SO_4_ for Pt_3_Co/N-CNT, Pt_3_Co/N-CNT_HT_, Pt_3_Co/S-CNT, Pt_3_Ni/N-CNT, Pt_3_Ni/N-CNT_HT_, Pt_3_Ni/S-CNT and the commercial Pt_3_Co/CB samples. For all the catalysts characterized, two small peaks at 0.14 and 0.24 V/RHE were observed, corresponding to the adsorption of H^+^ on the (110) and the (100) crystallographic faces of platinum, respectively [62]. Figure 4c,d represents the current–potential curves obtained by RRDE experiments in O_2_-saturated electrolyte at 900 rpm. These voltammograms are used to measure the ORR specific current density at 0.9 V/RHE. All the RRDE results are shown in Table 4.

**Table 4:** Electrochemical surface area (ECSA), current density and *i*_K_ at 0.9 V/RHE for Pt_3_M catalysts using RRDE experiments.

Sample	Pt loading (µg_Pt_ cm^−2^)	ECSA (m^2^·g_Pt_^−1^)	Current density at 0.9 V/RHE (mA·cm^−2^)	Kinetic current at 0.9 V/RHE (mA·cm^−2^)	Specific activity at 0.9 V/RHE (A/m^2^_Pt_)	Mass activity at 0.9 V/RHE (A·g_Pt_^−1^)

Pt_3_Co/CB	100	22.3	1.22	1.96	0.88	19.6
Pt_3_Co/N-CNT	106	55.5	2.12	5.33	0.91	50.3
Pt_3_Co/N-CNT_HT_	100	34.9	1.29	1.97	0.56	19.7
Pt_3_Co/S-CNT	99	23.1	1.30	2.47	1.08	25.0
Pt_3_Ni/N-CNT	106	34.7	1.85	3.49	0.95	32.9
Pt_3_Ni/N-CNT_HT_	106	46.4	1.66	3.09	0.63	29.1
Pt_3_Ni/S-CNT	94	5.6	0.58	0.74	1.41	7.9

The electrochemical surface area (ECSA) of the Pt_3_Co/N-CNT and Pt_3_Ni/N-CNT catalysts were higher than that of Pt_3_Co/CB (NP size ≈5 nm), probably due to the smaller size of the NPs (NP size ≈2 nm). These values (23–55 m^2^·g^−1^) are consistent with values reported in the literature for PtCo and PtNi catalysts on CNT [31,63–66], but showing larger particle size. These values are also lower than that obtained for Pt_3_Co particles with a slightly larger diameter (3.2–4.2 nm) (but on other carbon supports) where the ECSA values between 50 and 74 m^2^·g^−1^ were reported [67–70]. We think that remaining traces of ionic liquid adsorbed on the NP surface could be at the origin of this phenomenon. Indeed, it is not uncommon to observe an ECSA loss for Pt/C catalysts after IL modification [27].

Furthermore, we recently showed that when CNTs are used as catalyst supports, they show a tendency to form aggregates, making the accessibility of the electrolyte difficult, which could lead to a Pt utilization of 40%. For Pt_3_Co/S-CNT catalysts (and particularly the Pt_3_Ni/S-CNT catalyst), a much lower ECSA was obtained.

The specific activity has been calculated for each catalyst at 0.9 V/RHE. It appears that this value is mainly driven by the ECSA. The catalyst with the lower ESCA presents a higher specific activity and the catalyst with the higher ECSA has the lowest specific activity. This effect has been previously reported in the literature [71]. Nevertheless, we need to mention that the relatively high thickness of our prepared electrodes enhances the O_2_ diffusion resistance, which might enhance the surface activity for a material with lower ECSA.

This highlights the influence of the carbon support on the performance obtained in RRDE measurement. With 25.0 A·g_Pt_^−1^, the activity measured for PtCo/S-CNT is slightly higher than that of the commercial reference. The value obtained for PtNi/S-CNT is significantly lower (7.9 A·g_Pt_^−1^). A possible explanation could be linked to the Pt_3_Ni NP–support interaction. As Pt_3_Ni catalysts on S-doped carbon materials have already shown good performance for the ORR [72–73], we suspect that the explanation arises from an excessive amount of carbon support during the measurement. Indeed, the measurements were made with an equivalent Pt loading on the electrode; however, the Pt loading in PtNi/S-CNT is rather low, which should have led to a thicker active layer during RRDE measurements. This could explain the low activity of this catalyst.

The two highest ECSAs were measured for Pt_3_Co/N-CNT and Pt_3_Ni/N-CNT_HT_ at 55.5 m^2^·g_Pt_^−1^ and 46 m^2^·g_Pt_^−1^, respectively. For the RRDE characterization, a thin film of the catalyst was formed at the surface of the working electrode. For each carbon support used in this study, there was a modification of the properties of the catalytic layer and certainly a strong change of the mass transfer limitation. The proton transport should be better at the surface of the N-CNT due to the nitrogen doping and the hydrophilic behavior of these carbon supports [74]. For both metals, the catalysts with the best NP distribution on the CNT walls presented the higher ECSA. In these cases, we can assume that the interactions between Nafion^®^, NPs and N-CNTs are optimum to allow for high activity for the ORR. It should be noted that all the catalysts synthesized on N-doped CNTs outperformed the commercial Pt_3_Co/CB (22.3 m^2^·g_Pt_^−1^ and 19.6 A·g_Pt_^−1^) material. The highest mass densities were calculated for Pt_3_Co/N-CNT, Pt_3_Ni/N-CNT and Pt_3_Ni/N-CNT_HT_, with 50.3 A·g_Pt_^−1^, 32.9 A·g_Pt_^−1^ and 29.1 A·g_Pt_^−1^, respectively. Different hypotheses could explain the excellent results for these two catalysts. First, the well-distributed NPs are the result of the good interaction between the transition metal and the nitrogen-doped CNT. It is known that high binding energies between the transition metal and the carbon support modify the electronic properties of the NPs and can facilitate the adsorption of the O_2_. However, XPS data (vide infra) do not support this hypothesis, since the binding energy of Pt 4f electrons is consistent with Pt(0) in these two catalysts. Another hypothesis could be the particle proximity effect. Speder et al. show the influence of the distance of the neighboring particles on the ORR activity [75]. In fact, catalysts with small inter-particles distance with no agglomeration display excellent activity. Computational investigations have shown that decreasing the inter-particle distances causes an overlap of the electrochemical double layer. This overlap could reduce the oxide coverage of the NPs and thus increase the activity of the catalyst. Moreover, the synergetic effect of a Pt_3_CO catalyst supported on Co containing N-doped carbon material has recently been demonstrated [76].

It is worth noting that the effect of support annealing is not the same for the PtCo and PtNi catalysts. On the non-heat-treated support N-CNT the activity order follows that expected from the literature for 2 nm NPs in H_2_SO_4_ electrolyte: Pt_3_Co > Pt_3_Ni [58]. However, this order is reversed for the N-CNT_HT_ support since the specific activity (SA) is 1.66 and 1.29 mA·cm^−2^ for Pt_3_Ni and Pt_3_Co catalysts, respectively. On Pt_3_Co catalysts, the support annealing induces a pronounced decrease of both the ECSA and SA, whereas on Pt_3_Ni catalysts, the annealing induces an increase of the ECSA and a slight decrease of the SA. It is thus demonstrated that the specific activity of Pt_3_Ni and Pt_3_Co electrocatalysts for the ORR is electrolyte- and particle-size-dependent, as is already known, but also support-dependent, at least for small (2 nm) nanoparticles. For such smaller NPs, it is plausible that a modification of the d-band center occurs upon modification of the support [77].

**Figure 5:**
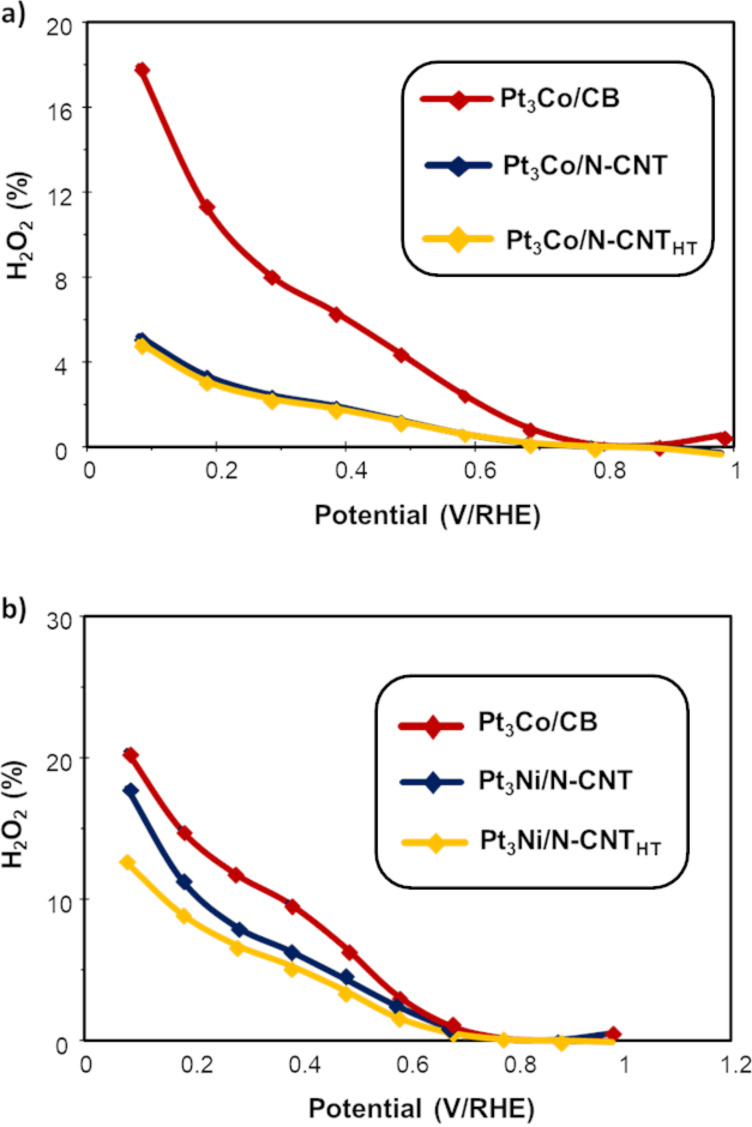
Percentage of H_2_O_2_ formation during O_2_ reduction of the a) Pt_3_Co and b) Pt_3_Ni catalysts.

The ORR selectivity is also a critical issue for a PEMFC catalyst. A well-known phenomenon of catalyst degradation is due to the formation of hydrogen peroxide near the electrolyte membrane [78]. Moreover, it was shown that in aqueous KNO_3_ solutions, nitrogen-doped carbon structures were active for ORR with lower H_2_O_2_ selectivity than Pt/C [79]. During the CV measurements in O_2_-saturated electrolyte, the amount of hydrogen peroxide produced during the ORR was monitored to compare its production for each catalyst. Figure 5 presents the hydrogen peroxide yield during the ORR for all the catalysts shown before.

First, between 1 and 0.7 V/RHE the fractions of H_2_O_2_ produced are below 1% for all catalysts. In this voltage region, the ORR occurs only through the four electron process. The H_2_O_2_ production is significantly higher at low potential, between 0.05 and 0.4 V/RHE, because of the oxygen reduction on carbon [80]. The amount of H_2_O_2_ produced by the reference Pt_3_Co/CB is higher than for all the Pt_3_Co samples and most of the Pt_3_Ni catalysts prepared in this study. Thus, the H_2_O_2_ production is not an obstacle for using these catalysts for PEMFC applications.

### Structural characterization of the best catalysts

**Figure 6:**
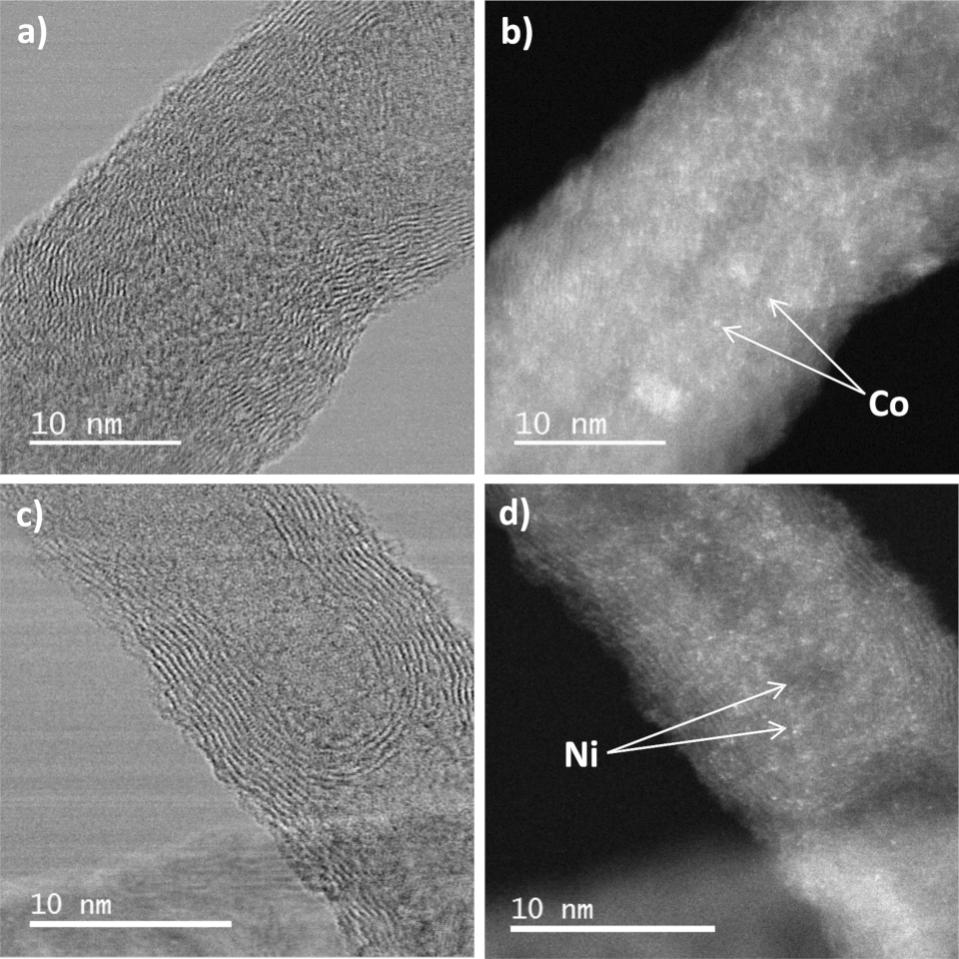
HRTEM images of a) Co/N-CNT and c) Ni/N-CNT_HT_; and STEM-HAADF images of b) Co/N-CNT and d) Ni/N-CNT_HT_.

For a better understanding of the structure of these catalysts, additional characterization was carried out on the Pt_3_Co/N-CNT and Pt_3_Ni/N-CNT_HT_ samples, which presented the best performance in the ORR. We first used HRTEM to analyze the product resulting from the first step of the catalyst preparation, i.e., the reduction of the cobalt and nickel salts. The HRTEM images of Co/N-CNT samples are depicted in Figure 6a,b.

First, no residual IL can be observed. Surprisingly, despite a high cobalt loading (52% w/w), Co NPs were also not observed on the surface of the N-CNTs. Instead, Co atoms (Figure 6a,b) and a few non-crystallized Co islands (Supporting Information File 1, Figure S7a,b) were identified using the scanning transmission electron microscopy/high-angle annular dark-field imaging (STEM-HAADF) technique. Energy-dispersive X-ray spectroscopy (EDX) analysis on the N-CNT surface or in the Co aggregates confirmed the presence of Co. The same analysis was made on the sample Ni/N-CNT_HT_. There also, despite the high Ni loading (48% w/w), no Ni NPs were observed (Figure 6c,d). STEM-HAADF analysis shed light into the presence of non-crystallized Ni at the surface of the carbon support. EDX analysis confirmed the presence of Ni at the surface of the N-CNTs.

**Figure 7:**
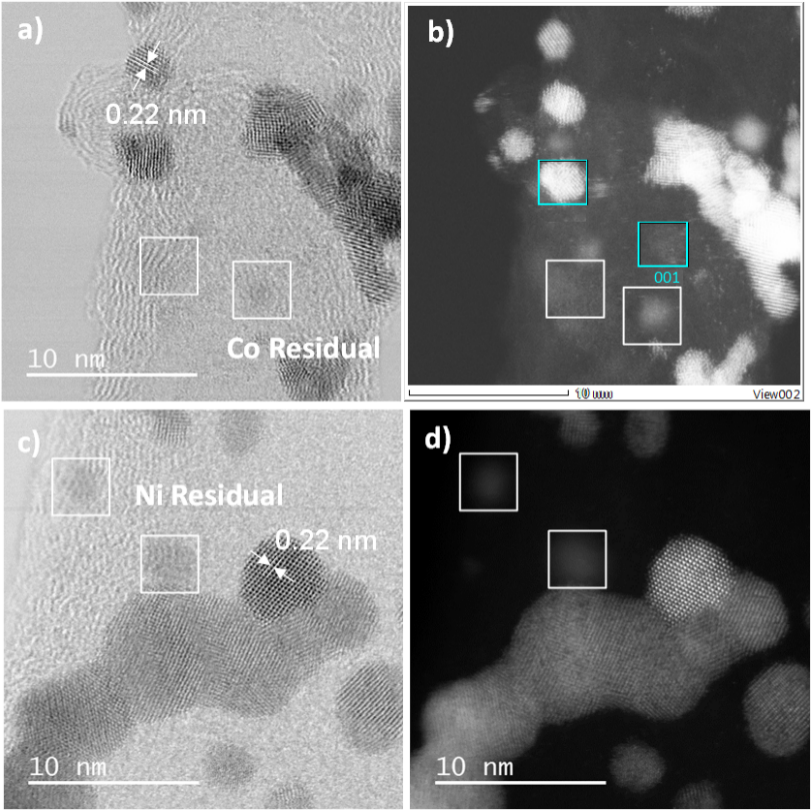
HRTEM images of a) Pt_3_Co/N-CNT and c) Pt_3_Ni/N-CNT_HT_, and STEM-HAADF images of b) Pt_3_Co/N-CNT and d) Pt_3_Ni/N-CNT_HT_.

HRTEM images of the bimetallic catalysts Pt_3_Co/N-CNT and Pt_3_Ni/N-CNT_HT_ do not evidence the formation of core–shell NPs (Figure 7). The interplanar distance of 0.22 nm was found for Pt_3_Co/N-CNT NPs and Pt_3_Ni/N-CNT_HT_ NPs (Figure 7), which is slightly smaller than the common distance found for d_111_ in Pt (0.23 nm) [81]. The slight contraction of the crystalline structure is probably due to the presence of Co (or Ni) atoms in the Pt structure. EDX spectra (Supporting Information File 1, Figure S8) reveal the presence of Co and Pt in the individual NPs (Figure 7b, 001 selected area) displaying a Pt_3_Co composition. The absence of Co NPs in the Co/N-CNT sample, and the composition and structure of the Pt_3_Co/N-CNTs, indicate that the Pt_3_Co/N-CNT is more likely an alloy than a core–shell structure. The presence of residual cobalt atoms or clusters on the CNT surface was also evidenced by STEM-HAADF images of Pt_3_Co/N-CNT (Figure 7b, 001 selected area). In the same way, the Pt_3_Ni composition was determine by EDX analysis for the sample Pt_3_Ni/N-CNT_HT_ (Supporting Information File 1, Figure S9). This sample also displays some residual nickel atoms or clusters.

**Figure 8:**
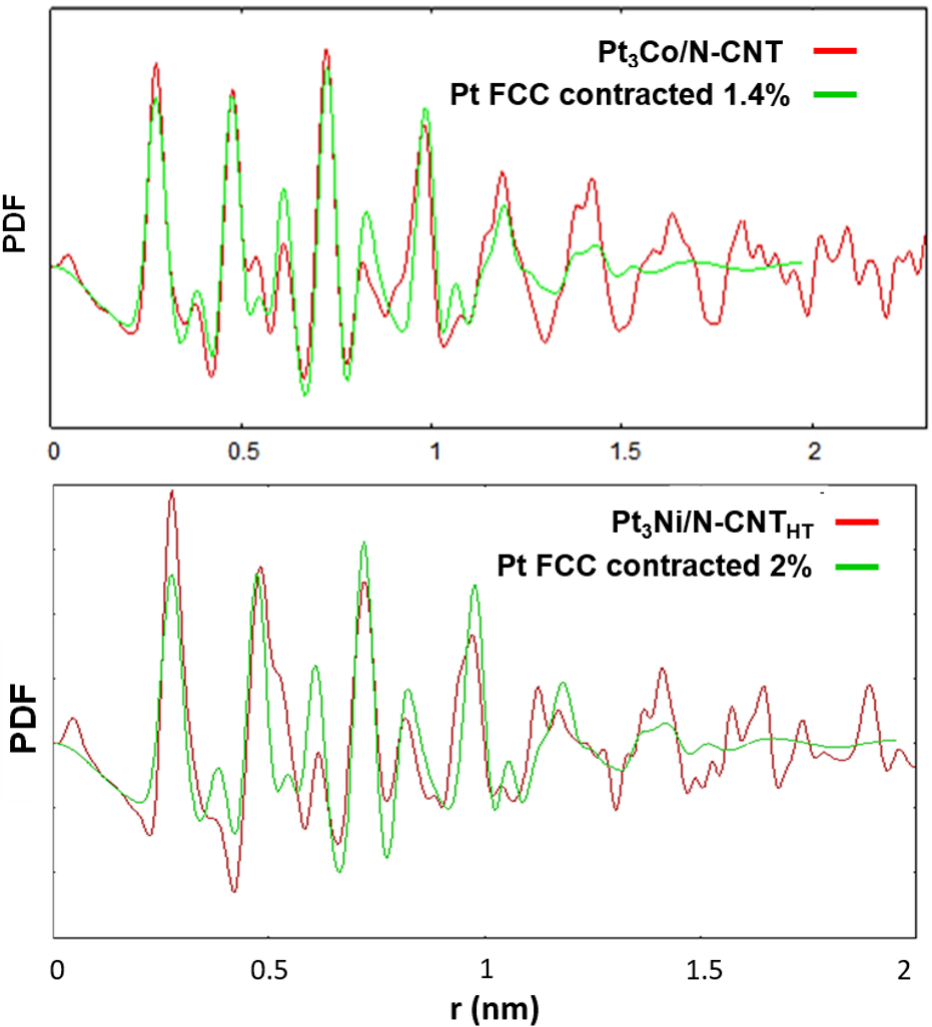
WAXS analysis – red: experimental PDF from a) Pt_3_Co/N-CNT and b) Pt_3_Ni/N-CNT_HT_, green: simulation from a spherical pure Pt model.

Interestingly, Co single atoms on nitrogen-doped carbon have been reported to be active for the ORR in acidic media [82–85], and Ni single atoms on nitrogen-doped carbon are known to be active for some electro-reduction reactions [86–87]. Thus, the involvement of these species in ORR cannot be discarded. Wide angle X-ray scattering (WAXS) analysis was performed on Pt_3_Co/N-CNT and Pt_3_Ni/N-CNT_HT_ (Figure 8). After corrections and taking a Fourier transform of the scattering data, the related pair distribution function (PDF) is well defined, with a low structural disorder. For Pt_3_Co/N-CNT, the coherence length is close to 2.3 nm, which gives a measurement of the average size of crystalline domains.

To accurately evaluate the actual cell parameter, thus the average composition, a simulation was performed from a model derived from pure Pt (spherical NPs, 2 nm in diameter).

To obtain a good agreement with the experimental data, a contraction factor of 1.4% was required, leading to a metallic bond length of 0.2721 nm, significantly shorter than in pure Pt (0.2760 nm – JCPDS 04–0802) but very close to the value in Pt_3_Co also crystallized in the Fm-3m system (0.2725 nm – JCPDS 29-0499) [88]. These results indeed point to an alloyed structure since in similar studies of core–shell NPs, the bond length obtained from the PDF was clearly related to the nature of the compact core [89]. The relatively poor agreement in amplitude for longer distances indicates that most crystalline domains are close to 2 nm but that some size and/or shape dispersion compared to the model is likely. The same conclusions can be made for the sample Pt_3_Ni/N-CNT_HT_. To obtain a good correlation with experimental data, a contraction factor of 2% was required. Concerning the NP size, most crystalline domains are close to 2 nm, which is consistent with the TEM result (1.86 nm).

XPS analysis was performed on the Pt_3_M/N-CNT catalysts in order to study the electronic structure of Pt, Co and Ni (Table 5 and below in Table 6).

**Table 5:** Binding energies and ratio of Pt and Co species obtained from XPS spectra.

Pt_3_Co/N-CNT	Species	Binding energy (eV)		Ratio (%)

		Pt 4f_7/2_	Pt 4f_5/2_	

Pt	Pt(0)	71.1	74.3	64.3
	PtO	72.1	75.2	12.8
	Pt(OH)_2_	73.1	76.6	22.9

		Co 2p_3/2_	Co 2p_1/2_	

Co	Co(0)	778.0	792.0	3.9
	CoO	779.8	785.0	60.4
	Co(OH)_2_	781.4	796.9	35.7

The Pt 4f region of the XPS spectrum is shown in Supporting Information File 1, Figure S10a. The spectrum was deconvoluted in three pairs of doublets (corresponding to Pt 4f_7/2_ and Pt 4f_5/2_). The first doublet at 71.08/74.3 eV corresponds to metallic Pt(0) [90]. The Pt surface is mainly in the metallic state (64.3 wt %). The doublets at 72.1/75.2 eV and 73.1/76.6 eV are assigned respectively to Pt(II) and Pt(IV) oxidation state species. The presence of Pt oxide and hydroxide is common for ultrafine Pt NPs [90–92]. The Co 2p region of the XPS spectrum (Supporting Information File 1, Figure S10b) reveals the presence of Co on the surface of the catalyst. The Co is mainly observed under oxidized state. In this area, peaks corresponding to CoO and Co(OH)_2_ can be observed at 779.8/785.0 eV and 781.4/796.9 eV, respectively. Considering the easy oxidation of cobalt in air, cobalt is always observed under these forms when PtCo alloys are studied [60,93–94]. As far as Co single atoms on nitrogen-doped carbon materials, it has been shown that nitrogen doping of the carbon provides sites for Co incorporation. On such supports, cobalt is usually found in the ionic state in a CoN_4_ environment. XPS characterization of Co single atoms on nitrogen-doped carbon has shown two peaks for Co at a binding energy of 781.1 and 796.2 eV [95]. However, the CoN_4_ single atoms are usually prepared by high temperature pyrolysis, and in our case, the synthesis is conducted at room temperature. It is very unlikely that CoN_4_ species will be formed under these conditions.

The results of the XPS analysis performed on Pt_3_Ni/N-CNT_HT_ revealed the species detailed in Table 6.

**Table 6:** Binding energies and ratio of Pt and Ni species obtained from XPS spectra.

Pt_3_Ni/N-CNT_HT_	Species	Binding energy (eV)		Ratio (%)

		Pt 4f_7/2_	Pt 4f_5/2_	

Pt	Pt(0)	71.2	74.4	60.1
	PtO	72.1	75.2	19.2
	Pt(OH)_2_	73.1	76.3	20.7

		Ni 2p_3/2_	Ni 2p_1/2_	

Ni	Ni(0)	852.8	869.9	4.4
	NiO	854.3	873.3	16.4
	Ni(OH)_2_	856.0	874.3	51.6
	NiOOH	857.6	874.6	27.6

The Pt 4f region of the XPS spectrum is shown in Supporting Information File 1, Figure S11a. The deconvolution shows three pairs of doublets (corresponding to Pt 4f_7/2_ and Pt 4f_5/2_). The first doublet at 71.2/74.4 eV corresponds to metallic Pt(0). The metallic state represents 60.1 wt % of the sample. The doublets at 72.1/75.2 eV and 73.1/76.6 eV are assigned to PtO and Pt(OH)_2_ oxidation state species, respectively. The Ni 2p region of the XPS spectrum is presented in Supporting Information File 1, Figure S11b. The deconvoluted spectrum indicates that most of the Ni is present under oxidized states. In this area, doublets corresponding to NiO, Ni(OH)_2_ and Ni(OOH) can be observed at 854.3/873.3 eV, 856.0/874.3 eV, and 857.6/874.6 eV, respectively [96]. The important concentration of oxidized species is generally observed when PtNi alloys are studied [96–97]. As far as Ni single atoms on nitrogen-doped carbon as concerned, oxidized species have also been reported [98].

Taking into account all these results, we propose the following mechanism for the synthesis of Pt_3_M NPs supported on N-CNTs. During the first step of the synthesis, the transition metal, M, will anchor the carbon surface in atomic form after reduction of the metal salt. Cobalt and nickel atoms are well-dispersed due to the use of the ILs. The addition of water for the hydrolysis of the NaBH_4_ may explain the partial oxidation of the transition metal and the presence of residual metal in the final product, as oxidized species cannot undergo the galvanic displacement. During the galvanic replacement, the M atoms in metallic form can react with the platinum salt. After washing the catalyst and removing of the IL, Pt_3_M NPs supported on N-CNTs are obtained with unreacted M atoms (and clusters) at the surface of the carbon support.

### Catalyst treatment: EDTA washing

The previous characterization revealed a large amount of unalloyed cobalt or nickel species on the support surface, which can be easily dissolved in acidic media, which then poisons the protonic group of the ionomer and the proton exchange membrane [5]. The impact of dissolved Co or Ni will be much more important in MEA configuration than in RRDE testing, specifically because the poisoned sulfonic groups could not be washed by liquid electrolyte in MEA, as it could be in RRDE setup. In order to minimize the impact of the treatment on both the catalyst and its support, we avoid the classically used acid leaching method [99], but preferred using and internally developed method, based on washing the material with a solution of ethylenediaminetetraacetic acid (EDTA). This consists of dispersing the Pt_3_Co/N-CNT or Pt_3_Ni/N-CNT_HT_ in a 0.1 M EDTA solution and using ultrasonication. For the Pt_3_Co/N-CNT catalyst, the solution become purple in a few seconds, indicating the fast and easy dissolution of the Co; while for Pt_3_Ni/N-CNT_HT_, the solution become blue after a few hours. One can suppose there is a better interaction between the Ni and the N-CNT_HT_ than the Co and the N-CNT. The catalysts were then filtered, washed with deionized water and dried for 24 hours at 80 °C.

**Figure 9:**
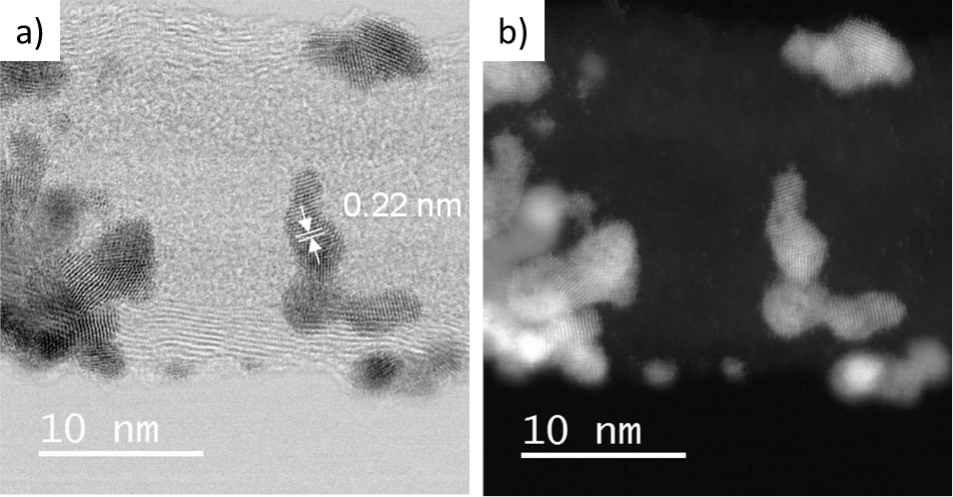
a) HRTEM images of Pt_3_Co/N-CNT and b) STEM-HAADF image of Pt_3_Co/N-CNT after washing with EDTA.

The same structural characterization was performed on the washed Pt_3_Co/N-CNT catalyst. The results of the XPS analysis are reported in Supporting Information File 1, Table S1. The data do not show significant variations of the Pt^(0)^/PtO/Pt(OH)_2_ and Co^(0)^/CoO/Co(OH)_2_-CoN_4_ values, except a low decrease of the metallic content for both Pt and Co after the EDTA washing. TEM and STEM-HAADF images are reported in Figure 9. The same interplanar distance of 0.22 nm for the NPs as before washing has been found by HRTEM (Figure 9a). The STEM-HAADF image in Figure 9b shows an important decrease of Co atoms clusters on the surface of the N-CNT, and the EDX analysis shows an important decrease of the Co amount after the washing (Supporting Information File 1, Figure S12).

WAXS analysis was also performed. The superimposition of the PDF after and before washing is shown in Supporting Information File 1, Figure S13. The results show that the nanoparticles are metallic and well crystalized but with a mean diameter slightly larger after washing (2.6 nm) than before washing (2.3 nm). In that case, a correction factor of 1.5% should be applied to obtain a good agreement between the experimental results and a simulation performed from a model derived from pure Pt (spherical NP, 2 nm in diameter). From the TEM images, the mean diameter was calculated to be 2.4 ± 1 nm. The inductively coupled plasma optical emission spectrometry (ICP-OES) analysis give a weight ratio of 3.8% for Co and 45.5% for Pt. These results show that this new washing procedure is efficient to removed unalloyed non noble metals. Its impact on the structure of the catalyst particles is limited and, in the case of Co, it takes just a few minutes. One can assume that the conditions are mild enough to avoid damaging the catalyst support. Nevertheless, after this treatment, the catalyst composition seems to be closer to Pt_4_Co than Pt_3_Co.

### Single cell testing and accelerated stress tests

**Figure 10:**
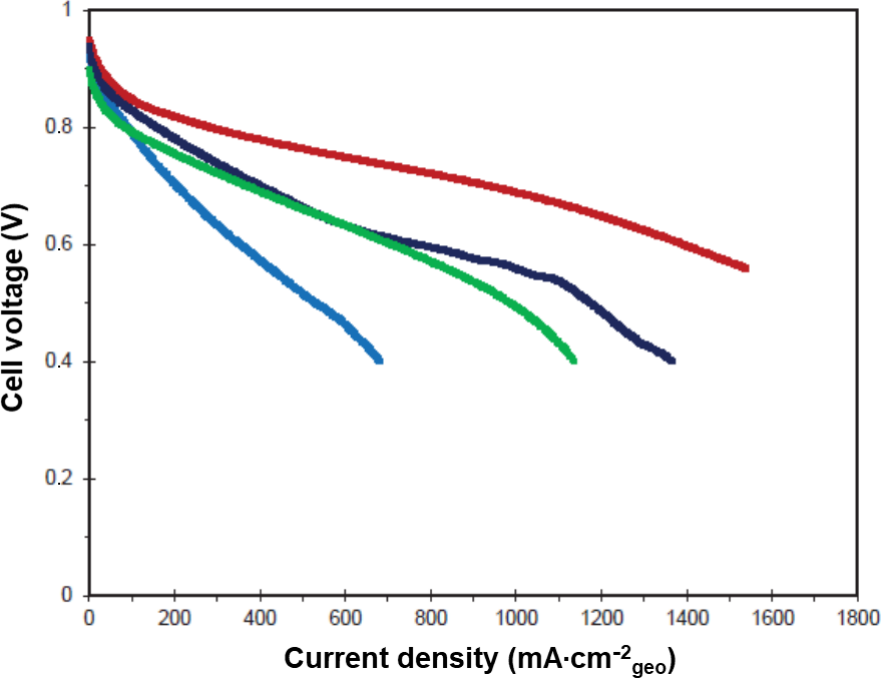
Polarization of Pt_3_Co/CB (red); Pt_3_Co/N-CNT (dark blue); Pt_3_Co/N-CNT_HT_ (light blue) and Pt_3_Ni/N-CNT_HT_ (green) recorded under air, *P*_inlet_ = 2.5 bar, *T* = 80 °C, St_H2_ = 1.2; St_Air_ = 3.5, RH_anode_ = 50%; RH_cathode_ = 30%.

First, the washed catalysts Pt_3_Co/N-CNT, Pt_3_Co/N-CNT_HT_ and Pt_3_Ni/N-CNT_HT_ were integrated into the MEA with a platinum loading of 0.26, 0.24 and 0.3 mg_Pt_/cm^2^, respectively. A reference MEA, integrating the commercial reference catalyst Pt_3_Co/CB with a loading of 0.3 mg_Pt_·cm^−2^, has been prepared and used as a reference. The polarization curves registered under air are reported in Figure 10. Focusing on the activation part of the curve, and as expected thanks to the RDE screening, Pt_3_Co/N-CNT is more active than Pt_3_Co/N-CNT_HT_ and Pt_3_Ni/N-CNT_HT_. The performance obtained under air was shown to achieve 574 mW·cm^−2^ for Pt_3_Co/N-CNT, 278 mW·cm^−2^ for Pt_3_Co/N-CNT_HT_ and 494 mW·cm^−2^ for Pt_3_Ni/N-CNT_HT_. When the Pt_3_Co/N-CNT is tested under pure O_2_ the power density obtained at 1.5 A·cm^−2^ is 996 mW·cm^−2^, which is higher than the results previously reported on an MEA integrating catalysts supported on CNTs [100–101].

The polarization curves recorded under O_2_ are shown in Supporting Information File 1, Figure S14. Even this performance is still lower than the performance of the reference MEA, 863 mW·cm^−2^ under air and 1118 mW·cm^−2^ under O_2_ at 1.5 A/cm^2^.

**Figure 11:**
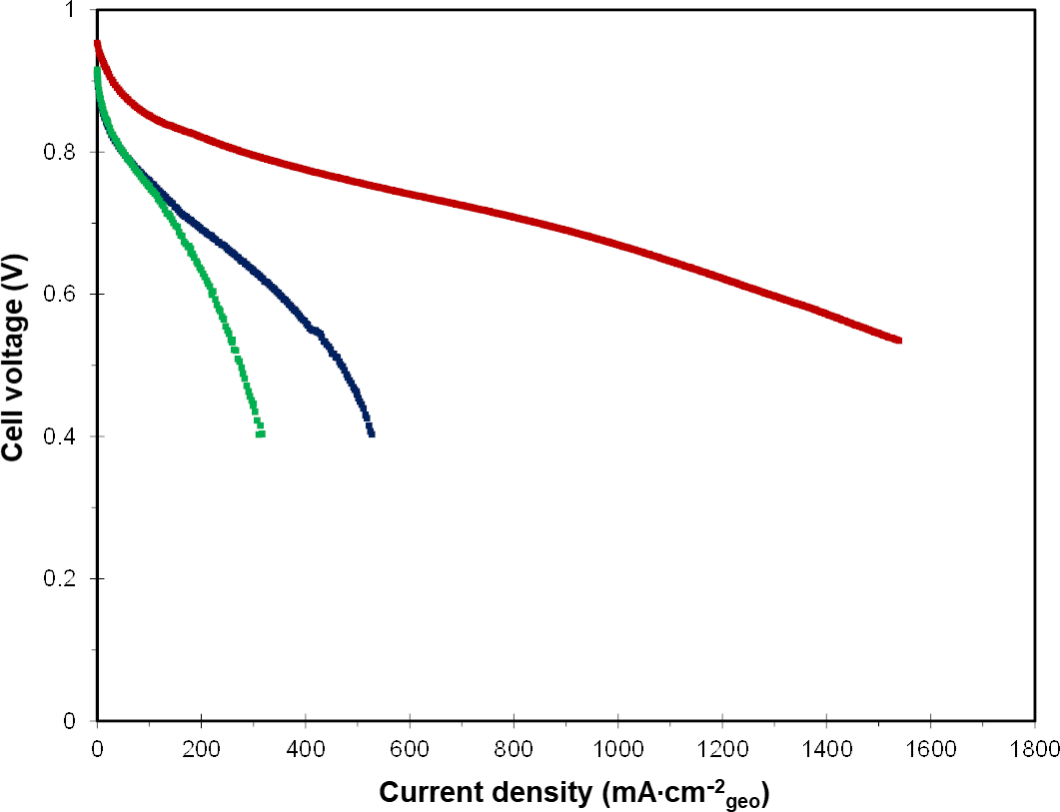
Polarization of Pt_3_Co/CB (red), unwashed Pt_3_Co/N-CNT (dark blue) and unwashed Pt_3_Ni/N-CNT_HT_ (green) recorded under air, *P*_inlet_ = 2.5 bar, *T* = 80 °C, St_H2_ = 1.2; St_Air_ = 3.5, RH_anode_ = 50%; RH_cathode_ = 30%.

To evidence the added value of the EDTA washing protocol, an MEA integrating the catalysts not treated with a solution of EDTA are shown in Figure 11. One can see that, even at very low current, the beneficial impact of the developed washing protocol is clear. We suspect that for the unwashed catalyst the non-alloyed Co or Ni could be leached during the ink preparation and then trapped by the ionomer.

Based on the quantitative analysis of the catalyst composition, it can be calculated that for the unwashed Pt_3_Co/N-CNT, 1.5 µmol of Co^2+^ is released into the ink, for each square centimeter of prepared MEA. As the cathode contain 20% weigh ratio of Nafion (equivalent weight: 1000), each square centimeter of cathodic active layer contains 0.18 µmol of sulfonic acid site. The anode is composed of 0.2 mg_Pt_·cm^−2^ and a Nafion content of 25%, which leads to 0.13 µmol of sulfonic acid site per square centimeter of MEA. According to the membrane thickness (20 µm) the Nafion density (1.02 g·mL^−1^), and disregarding the impact of the reinforcement, it can be considered that 1 cm^2^ of HP membrane contains around 2 µmol of sulfonic acid site. Therefore, the total amount of sulfonic acid group in the MEA is around 2.31 µmol·cm^−2^_geo_, which is less than two times the theoretical amount of released Co. As one Co^2+^ cation can neutralize two sulfonic acid groups, the proton transport in the MEA integrating unwashed catalyst is almost impossible, which explains such low performance, even at low current.

**Figure 12:**
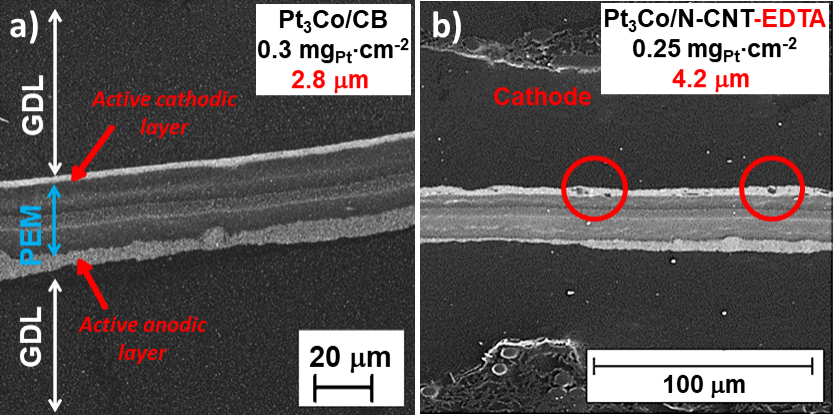
SEM micrographs of MEA sections prepared with Pt_3_Co/CB at 0.3 mg_Pt_·cm^−2^ and Pt_3_Co/CNT-N at 0.25 mg_Pt_·cm^−2^.

Electrochemical impedance spectra (EIS) were collected on single cell containing an MEA based on Pt_3_Co/N-CNT and Pt_3_Co/CB at 0.1 A/cm^2^, under air and in the same conditions as the polarization experiments. The high frequency resistance was 4.6 and 3.2 mΩ, respectively (see Supporting Information File 1, Figure S15). The higher value for the MEA integrating the catalyst supported on CNTs might be due to worse dispersion of the ionomer into the active layer and worse interface of the active layer with the membrane than with the reference catalyst. Indeed, it is known that CNTs tend to form aggregates and worsen dispersion than Vulcan-like carbon black. This was also observed in SEM images of the prepared MEA (Figure 12).

**Figure 13:**
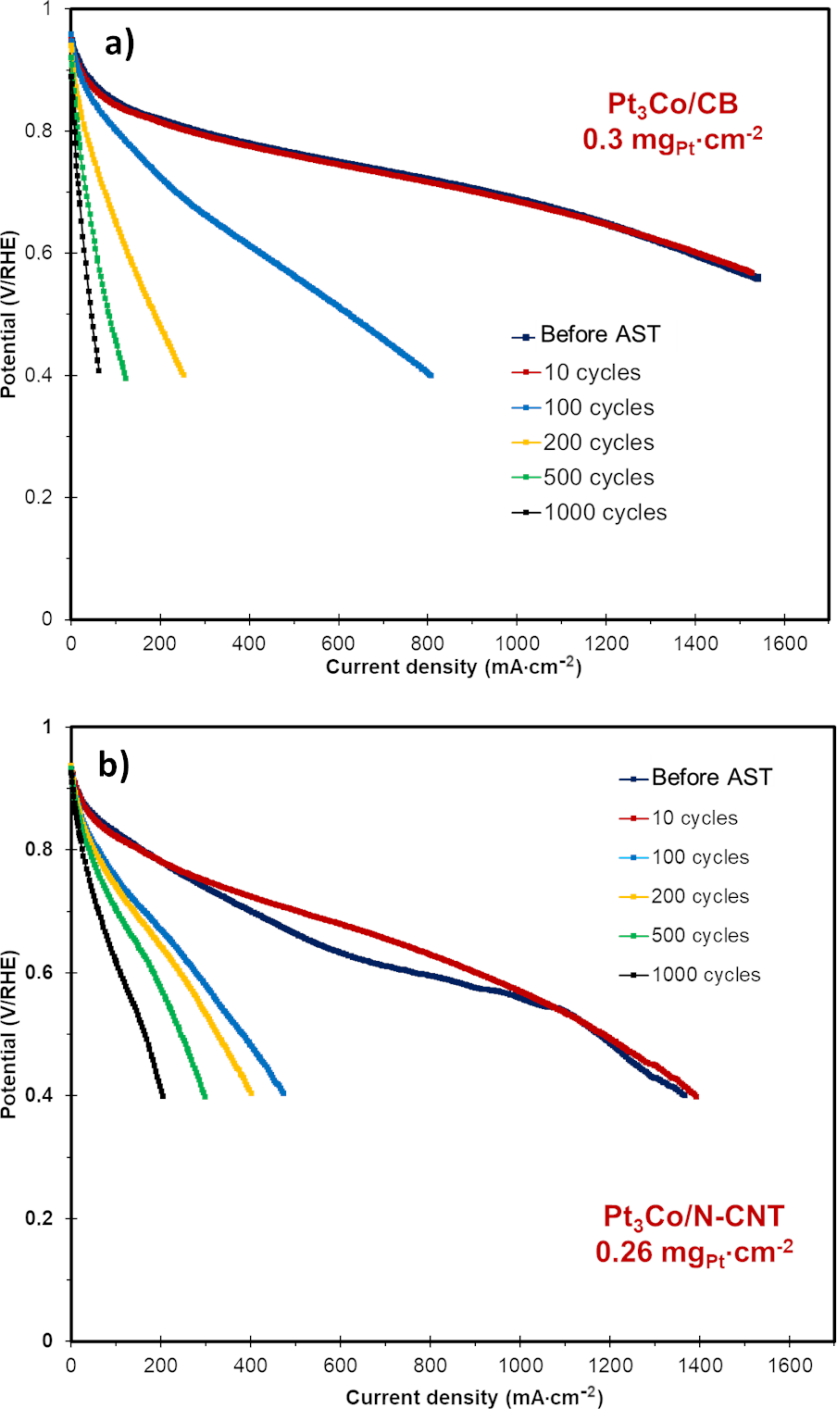
Polarization curves performed on MEAs integrating: a) Pt_3_Co/CB; and b) Pt_3_Co/N-CNT. Dark blue: after conditioning, red: after 10 cycles, light blue: after 100 cycles, yellow: after 200 cycles, green: after 500 cycles and black: after 1000 cycles.

Next, the catalyst support stability was evaluated using an AST for this purpose [102]. The evolution of the polarization registered during the AST is shown is Figure 13 for both the reference MEA and the MEAs integrating the Pt_3_Co/N-CNT catalysts.

It can be observed that the degradation rate for the catalyst supported on N-CNTs is slower than the reference commercial catalyst. Indeed, even if the beginning-of-life performance of the reference MEA is better, after only 200 cycles, the performance of the Pt_3_Co/N-CNT MEA is then better. This validates the robustness of the synthesized catalyst.

## Conclusion

In summary, a series of undoped, S- and N-doped CNTs have been synthetized by c-CVD. These carbon materials have been used together with an ionic liquid as a support structure for bimetallic Pt_3_Co and Pt_3_Ni NPs by a transmetalation method. The nature of the support significantly produced the NP distribution on the supports. For Pt_3_Co catalysts, a high metal loading (>45% w/w) and small NP size (<2 nm) was obtained on the support presenting the higher nitrogen content. Surprisingly, for Pt_3_Ni catalysts, a higher loading and smaller particle size was obtained on N-doped CNTs that were submitted to a high temperature treatment, which decreased the nitrogen content. HRTEM, WAXS and XPS analyses of the Pt_3_Co and Pt_3_Ni samples revealed that bimetallic Pt_3_M NP alloys were obtained with this method. Residual cobalt or nickel atoms were also present on the N-CNT surface. The experimental results showed enhanced catalytic performance for catalysts prepared with N-CNTs compared to the S-CNTs and commercial Pt_3_Co/CB catalysts. The best results were obtained for Pt_3_Co that used the more hydrophilic supports (N-CNT), and for Pt_3_Ni with the heat-treated N-CNT_HT_ supports. Interestingly, our study shows that the catalytic performance is support-dependent. For the N-CNT support, the SA followed the trend Pt_3_Co > Pt_3_Ni, in accordance with literature reports; whereas for N-CNT_HT_, the SA followed the opposite order.

The use of Pt_3_M/N-CNT catalysts results in the reduction in the quantity of H_2_O_2_ produced during the ORR compared to the commercial Pt_3_Co/CB catalyst. After ex situ validation of the catalyst, the treatment with EDTA solution to remove unalloyed non noble metals (Co or Ni) was employed. The electrochemical characterization of the MEA containing washed and unwashed catalysts validated this new protocol. The ageing tests (AST), characterizing the catalyst support degradation, showed better resistance toward degradation of the Pt_3_Co/N-CNT than the reference Pt_3_Co/CB catalyst. Beyond these results, our future works will focus on the end-of-life analysis on the aged MEA and on the integration of catalysts supported on CNTs in the active layer to increase the beginning-of-life performance of the MEA.

## Experimental

The metal precursors used for the syntheses were purchased from Strem Chemicals Inc., the ionic liquid was purchased from Solvionic, and the other chemicals were purchased from Sigma-Aldrich. All operations were carried out under argon atmosphere using standard Schlenk techniques or in an MBraun glovebox. For comparison purpose, the commercial catalyst Pt_3_Co/CB consists of 6 wt % Co, 46.7 wt % Pt on carbon black from Tanaka Kikinzoku Kogyo (reference TEC36V52) with NPs of around 5 nm.

### CNT synthesis and functionalization

CNTs were grown using a catalytic-CVD process: ethylene as a carbon source, acetonitrile as a nitrogen/carbon source and thiophene as a sulfur/carbon source were decomposed at 650 °C on a Fe/Al_2_O_3_ catalyst in a vertical oven to produce CNTs and N-CNTs. First, the catalyst was reduced under argon/hydrogen (Ar/H_2_ (1.5/1): 375 mL·min^−1^) during 30 min at 650 °C. Undoped structures (called CNTs) were prepared from ethylene/H_2_ (375 mL·min^−1^ (1.5/1)) mixtures, N-doped structures (called N-CNTs) from acetonitrile/Ar/H_2_ (375 mL·min^−1^, Ar/H_2_ (1.5/1) bubbling through acetonitrile at 35 °C, 0.19 bar vapor pressure), and S-doped structures (called S-CNTs) according to a published procedure [103]. All CNTs were purified with a refluxing mixture of H_2_O/H_2_SO_4_ (50/50 v/v) for 3 h to remove the catalyst. The annealing of the N-CNTs was carried out at 1000 °C. 0.5 g of sample was placed in the chamber of a horizontal oven under Ar (200 mL·min^−1^). The annealing temperature was reached with a rate of 10 °C·min^−1^ and held for 2 h. Finally, the sample was cooled down to room temperature under Ar to obtain N-CNT_HT_.

#### Catalyst preparation

The Pt_3_Co/CNT catalysts were prepared following a modified procedure reported elsewhere [23]. Here, the ionic liquid, 1-butyl-3-methylimidazolium bis(trifluoromethanesulfonyl)imide ([bmim][Tf_2_N]) was used as a stabilizer in order to increase the interaction between the carbon support and the metallic salt and favor the formation of small nanoparticles. 2.04 mmol of CoCl_2_·6H_2_O were dissolved in 60 mL of ethanol and added in a 30 mL ethanol solution containing 0.2 g of CNTs and 0.48 mmol of [bmim][Tf_2_N]. The reaction mixture was sonicated for 20 min and then stirred vigorously. A freshly prepared NaBH_4_ solution in ethanol (0.15 mol·L^−1^) was added to the reaction mixture and allowed to react for 30 min. Afterwards, 100 mL of deionized water were added and the suspension was stirred for 3 h. Next, 1.27 mmol of K_2_PtCl_4_ was dissolved in 60 mL of deionized water added to the solution. After stirring overnight, the solution was filtered; the product was washed with ethanol and deionized water, and finally dried at 80 °C. The Pt_3_Ni/CNT catalysts were prepared using the same procedure using NiCl_2_·6H_2_O as the metal precursor.

#### Characterization

The samples were characterized using transmission electron microscopy (TEM, JEOL, JEM-1011 at 100 kV) and high-resolution TEM (HRTEM, JEM-ARM200F Cold FEG, STEM-EDX CENTURIO-X, GATAN Gif quantum ER), chemical analysis (CHN Perkin Elmer elemental analyzer), Raman spectroscopy at 633 nm (SmartsSPM-1000 AIST-NT) and thermal analysis under air (thermobalance Perkin Elmer Diamond TG). The textural characterization (BET surface area, *S*_BET_) of the material was evaluated by N_2_ adsorption–desorption analysis at −196 °C using a Quantachrome autosorb device. X-ray photoelectron spectroscopy (XPS) was performed on a ThermoScientific XPS K-alpha apparatus, which operated with an achromatized Mg K source (1253.6 eV). Pt and Co loadings were determined by inductively coupled plasma optical emission spectrometry (ICP-OES) analysis (Thermo Scientific, ICAP 6300 instrument). Wide angle X-ray scattering (WAXS) measurements were performed on a diffractometer dedicated to pair distribution function (PDF) analyses: graphite-monochromatized molybdenum radiation (0.07169 nm), solid state detection and low background setup. The samples were sealed in Lindemann glass capillaries (diameter 1.5 mm).

The MEA cross-sections were prepared by first cutting MEA samples (8 × 8 mm^2^) and embedding them in epoxy resin. Then, the MEA cross-sections were prepared by mechanical polishing until a mirror-like surface was achieved and were observed using a Zeiss FEG-SEM LEO1530.

### Electrochemical measurements

#### RRDE measurements

The electrochemical properties of the prepared catalysts were investigated in a three-electrode system in 0.5 M H_2_SO_4_ solution at room temperature using a RRDE. A saturated mercury sulfate electrode (MSE, Bioanalytical system Inc., RE-2C) was used as the reference electrode and a platinum wire as the counter electrode (CE). All the potentials are presented to the reversible hydrogen electrode (RHE). The measurements were carried out using a BIOLOGIC VSP potentiostat. The used RRDE is the model AFE7R9GCPT from PINE research, and the disk is glassy carbon with an area of 0.2475 cm^2^. The ring is in Pt with a collection factor of 37%. The electrode is polished with 1 µm diamond paste and 0.05 µm alumina paste before use. The RRDE electrodes were prepared from a suspension of PtCo/CNT catalyst in 4 mL of isopropanol/DI water/Nafion^®^ dispersion (type D-2020 from Dupont Fluoroproduct, 20% Nafion^®^ dissolved in aliphatic alcohol) (80/19.5/0.5), and sonicated for 30 min. 30 µL of the prepared ink was deposited three times onto the polished glassy carbon disk electrode. A thin catalytic layer with a Pt loading of 100 µgPt/cm^2^ was obtained after evaporation of the solvent under air at room temperature. The electroactive surface area (ECSA) was calculated from the second cyclic voltammetry (CV) curves using an electrolyte saturated with N_2_ at 5 mV·s^−1^, from 0.04 to 1.08 V/RHE. The ECSA was estimated by integrating the current in the hydrogen desorption region between 0.04 and 0.4 V/RHE on the positive-going potential scan, corrected by the double layer current at 0.4 V/RHE and assuming 210 µC·cm^−2^_(Pt)_ (Table 5). The activity was measured on the cyclic voltamograms using an electrolyte saturated with O_2_ at 5 mV·s^−1^ from 0.04 to 1.08 V/RHE fixing the rotation speed to 900 rpm The rotation is controlled by a PINE Research Instrumentation/model AFMSRCE device. The current density is normalized to the geometric surface area of the glassy carbon (0.247 cm^2^). The kinetic current is calculated following the equation:


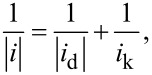


where *i* is the measured current at 0.9 V/RHE under O_2_ and corrected by a reference measurement made under N_2_ at the same potential and *i*_d_ is the measured current at 0.4 V/RHE under O_2_ and corrected by a reference measurement made under N_2_ at the same potential. There is no internal resistance correction.

The mass activity is finally obtained using the kinetic current density and the mass of the Pt loaded on the electrode. While the ORR occurs on the disk, the ring current was recorded, corresponding to H_2_O_2_ oxidation. This method was already reported elsewhere [104].

#### Single cell testing and accelerated stress tests

The single cell tests were performed using a Green Light (GL-40) test station. A graphite monopolar plate with a single serpentine flow field and 1 mm channel and landing dimensions were used. The active areas were 25 cm^2^. The polarization curves were recorded under current control, from open-circuit voltage (OCV) to high current with a ramping of 2 A·min^−1^. The polarization curves were recorded at 80 °C, with a pressure inlet of 2.5 bar on both sides, a stoichiometry of 1.2 at the anode side and 3.5 at the cathode side when air was used, or 5 when pure O_2_ was used. The relative humidity was managed by boilers and the values are 50% at the anode and 30% at the cathode. The conditioning is performed under air by maintaining the cell voltage at 0.5 V for 8 h, the operating conditions are similar but the relative humidity fixed at 100% on both sides.

For the ASTs, the cell was maintained at 80 °C, and the inlet pressure was 2.5 bar. The anode and cathode were fed with H_2_ and N_2_, respectively, both with 100% relative humidity. The cell voltage was controlled by a Gamry Instruments reference 3000 potentiostat. The cell voltage was cycled between 1.0 V and 1.5 V with a scan rate of 500 mV·s^−1^. The polarization curves were registered under air after 10, 100, 200, 500 and 1000 cycles. The MEAs were prepared by hot pressing gas diffusion electrodes on a reinforced HP Nafion^®^ membrane. The cathodes were prepared by manually spraying the cathodic active layer on the gas diffusion layer (GDL) (SGL-sigracet^®^ 24 BC). The used ionomer was Nafion^®^ D2020, its dry extract in the active layer is 20% for the synthesized catalysts and 25% for the commercial catalyst. The anode catalyst is Pt/CB 50% weight ratio from TKK, the loading of the anode is 0.2 mg_Pt_/cm^2^ and the gas diffusion electrode was prepared by screen printing.

## Supporting Information

File 1 Additional experimental data.
