# Supplementary material for: Alloyed Pt3M (M = Co, Ni) nanoparticles supported on S- and N-doped carbon nanotubes for the oxygen reduction reaction
Source: Beilstein J Nanotechnol. 2019 Jun 21;10:1251–69. doi: 10.3762/bjnano.10.125 (PMC6604734; doi:10.3762/bjnano.10.125)
Supplement: File 1 — Additional experimental data. [file Beilstein_J_Nanotechnol-10-1251-s001.pdf]

BEILSTEIN JOURNAL OF NANOTECHNOLOGY

Supporting Information

for

Alloyed Pt_3M (M = Co, Ni) nanoparticles supported on S- and N-doped carbon nanotubes for the oxygen reduction reaction

Stéphane Louisia, Yohann R. J. Thomas, Pierre Lecante, Marie Heitzmann, M. Rosa Axet, Pierre-André Jacques and Philippe Serp

Beilstein J. Nanotechnol. **2019,** *10,* 1251–1269. doi:10.3762/bjnano.10.125

Additional experimental data

License and Terms: This is a supporting information file under the terms of the Creative Commons Attribution License (http://creativecommons.org/licenses/by/4.0). Please note that the reuse, redistribution and reproduction in particular requires that the authors and source are credited.
The license is subject to the *Beilstein Journal of Nanotechnology* terms and conditions: (https://www.beilstein-journals.org/bjnano)

This file includes:

Table S1: Binding energies and ratio of Pt and Co species obtained from XPS spectra after washing with EDTA solution.

Figure S1: TEM micrographs of: a) CNT; b) N-CNT; c) S-CNT, and d) N-CNT_{HT}. (Scale bar = 50 nm).

Figure S2: Raman spectra at an incident laser energy of 2.33 eV (532 nm) of: a) CNT; b) N-CNT; and c) S-CNT.

Figure S3: Deconvolution of XPS spectra for S-CNT S_{2p}.

Figure S4: Deconvolution of XPS spectra for a) N-CNT N_{1s}, b) N-CNT S_{2p}, and c) N-CNT_{HT} N_{1s}.

Figure S5: a) TEM images of Pt_3Co/CNT synthetized with hexadecyl trimetyl ammonium bromide as stabilizer, and size distribution for the catalyst (the mean particle size is 1.90 ± 0.77 nm). b) TEM images of Pt_3Co/CNT synthetized with [bmim][Tf_2N] as stabilizer, and size distribution for the catalyst (the mean particle size is 2.28 ± 0.82 nm).

Figure S6: Size distribution for a) Pt_3Co/N-CNT, b) Pt_3Co/N-CNT_{HT}, c) Pt_3Co/S-CNT, d) Pt_3Ni/N-CNT, e) Pt_3Ni/N-CNT_{HT} and f) Pt_3Ni/S-CNT.

Figure S7: HRTEM images of a) Co/N-CNT and c) Ni/N-CNT_{HT}; and STEM-HAADF images of b) Co/N-CNT and d) Ni/N-CNT_{HT}.

Figure S8: a) HREM image and b) STEM-HAADF micrograph of Pt_3Co/N-CNT and EDX spectrum of the selected area.

Figure S9: a) STEM-HAADF micrograph of Pt_3Co/N-CNT and b) EDX spectrum of the selected area.

Figure S10: Deconvolution of XPS spectra for Pt_3Co/N-CNT a) Pt_{4f}, and b) Co_{2p}.

Figure S11: Deconvolution of XPS spectra for Pt_3Ni/N-CNT_{HT} a) Pt_{4f}, and b) Ni_{2p}.

Figure S12: Results of the EDX analysis on Pt_3Co/N-CNT a) before and b) after washing with EDTA solution.

Figure S13: WAXS analyses – a) diffractogram from Pt_3Co/N-CNT before (red) and after (green) washing with EDTA and simulation for a spherical pure model; b) experimental PDF from Pt_3Co/N-CNT before (red) and after (green) washing with EDTA.

Figure S14: Polarization curves of MEA based on Pt_3Co/CB (red) and Pt_3Co/N-CNT (dark blue); recorded under O_2, P_{inlet} = 2.5 bar, T = 80 °C, St_{H2} = 1.2; St_{O2} = 5, RH_{anode} = 50%; RH_{cathode} = 30%.

Figure S15: Nyquist diagrams of EIS for MEA based on Pt_3Co/CB (red) and Pt_3Co/N-CNT (dark blue) registered under air, P_{inlet} = 2.5 bar, T = 80 °C, St_{H2} = 1.2; St_{Air}= 3.5, RH_{anode} = 50%; RH_{cathode} = 30%.

Table S1: Binding energies and ratio of Pt and Co species obtained from XPS spectra after washing with EDTA solution.

Pt_3Co/N-CNT	Species	Binding energy (eV)		Ratio (atom %)
		**Pt 4f$_{7/2}$**	**Pt 4f$_{5/2}$**	
**Pt**	Pt(0)	71.1	74.3	57.8
	PtO	72.1	75.2	15.8
	$Pt(OH)_2$	73.1	76.4	26.4
		**Co 2p$_{3/2}$**	**Co 2p$_{1/2}$**	
**Co**	Co(0)	778.5	792.5	16.4
	CoO	780.0	794.5.0	48.9
	$Co(OH)_2/CoN_4$	781.8	796.4	34.9

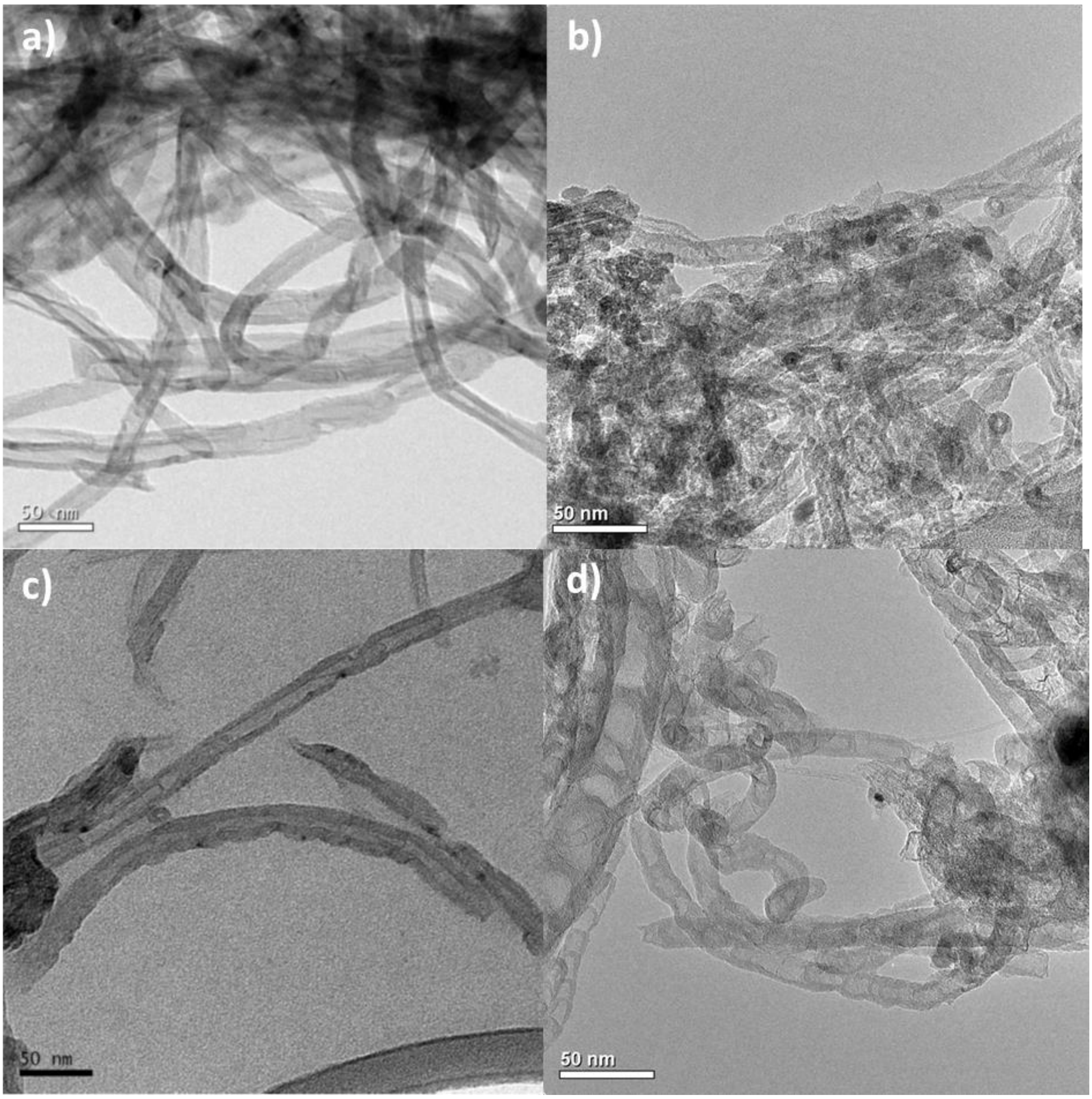

Figure S1: TEM micrographs of: a) CNT; b) N-CNT; c) S-CNT, and d) N-CNT$_{HT}$. (Scale bar = 50 nm).

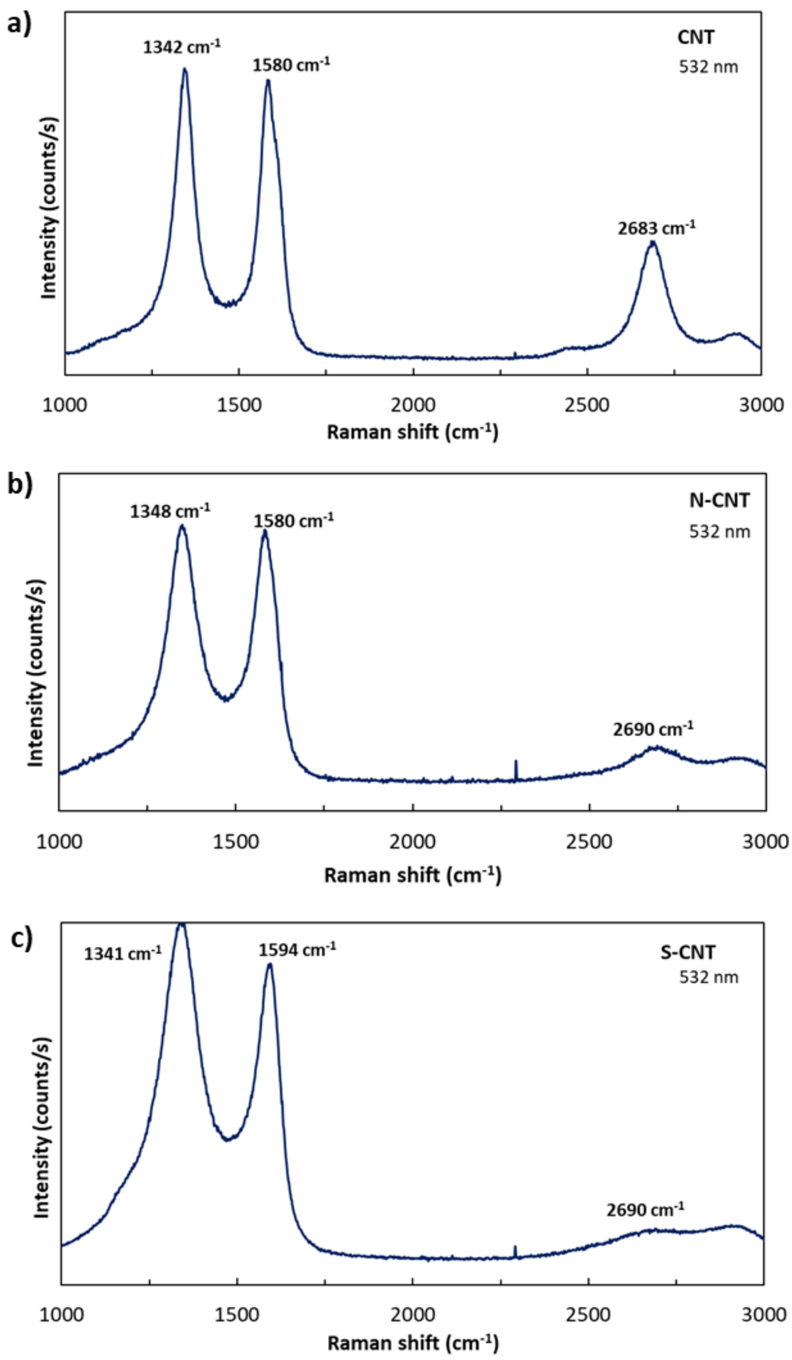

Figure S2: Raman spectra at an incident laser energy of 2.33 eV (532 nm) of: a) CNT; b) N-CNT; and c) S-CNT.

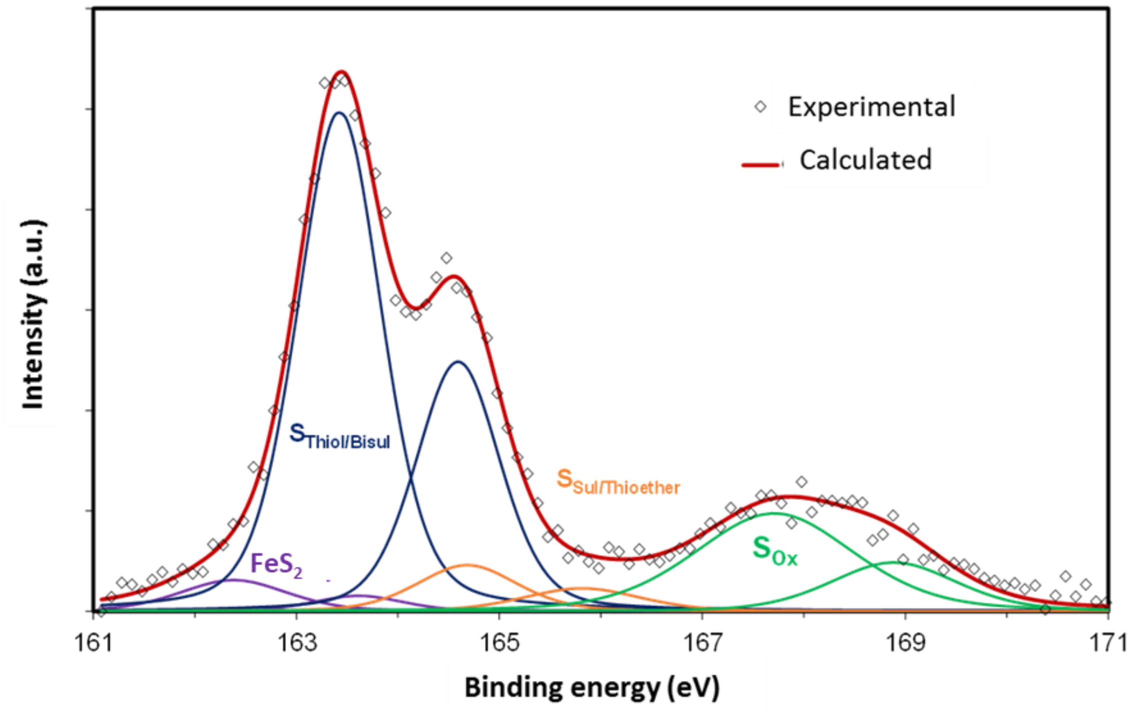

Figure S3: Deconvolution of XPS spectra for S-CNT S_{2p}.

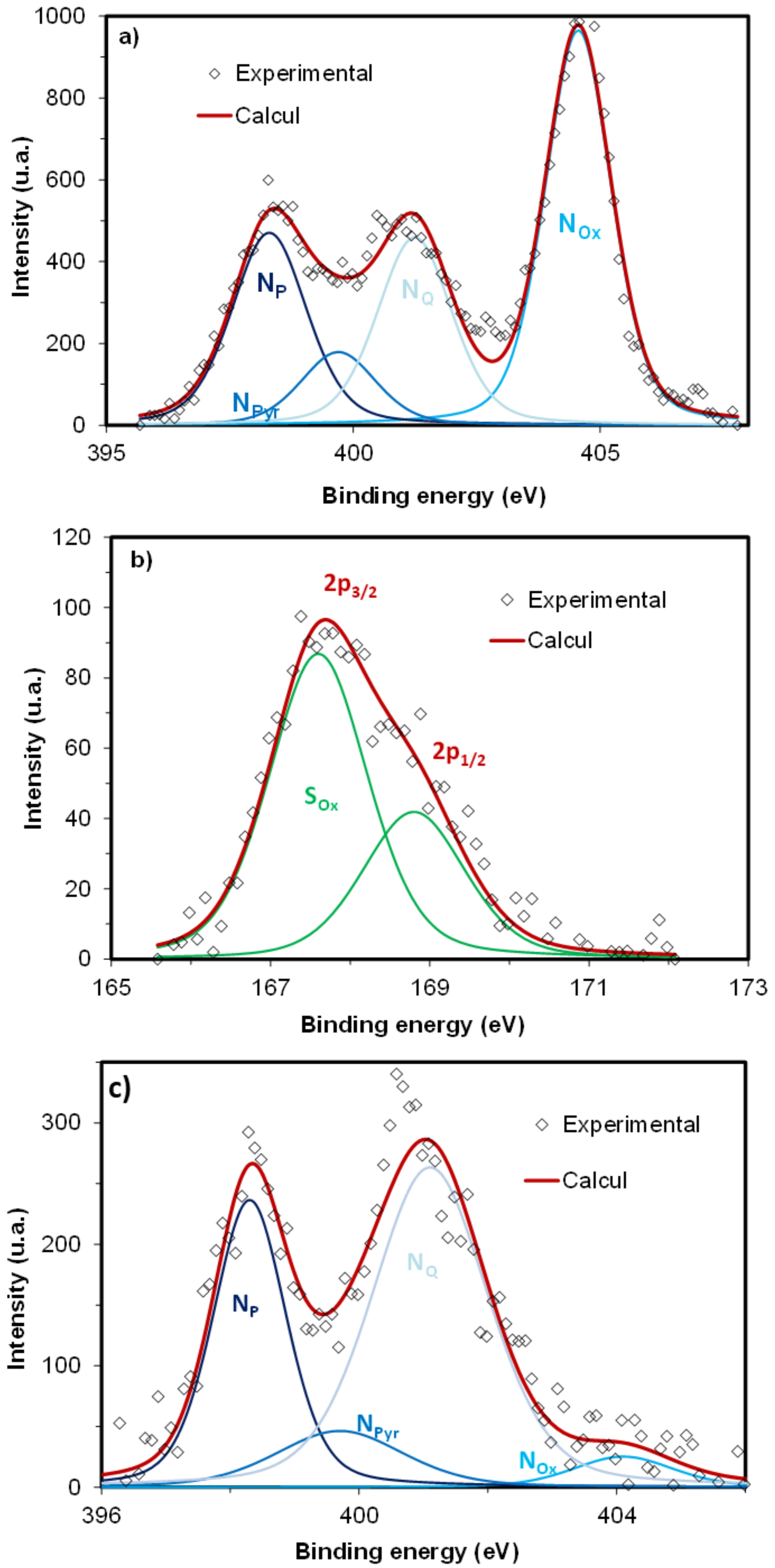

Figure S4: Deconvolution of XPS spectra for a) N-CNT N_{1s}, b) N-CNT S_{2p}, and c) N-CNT_{HT} N_{1s}.

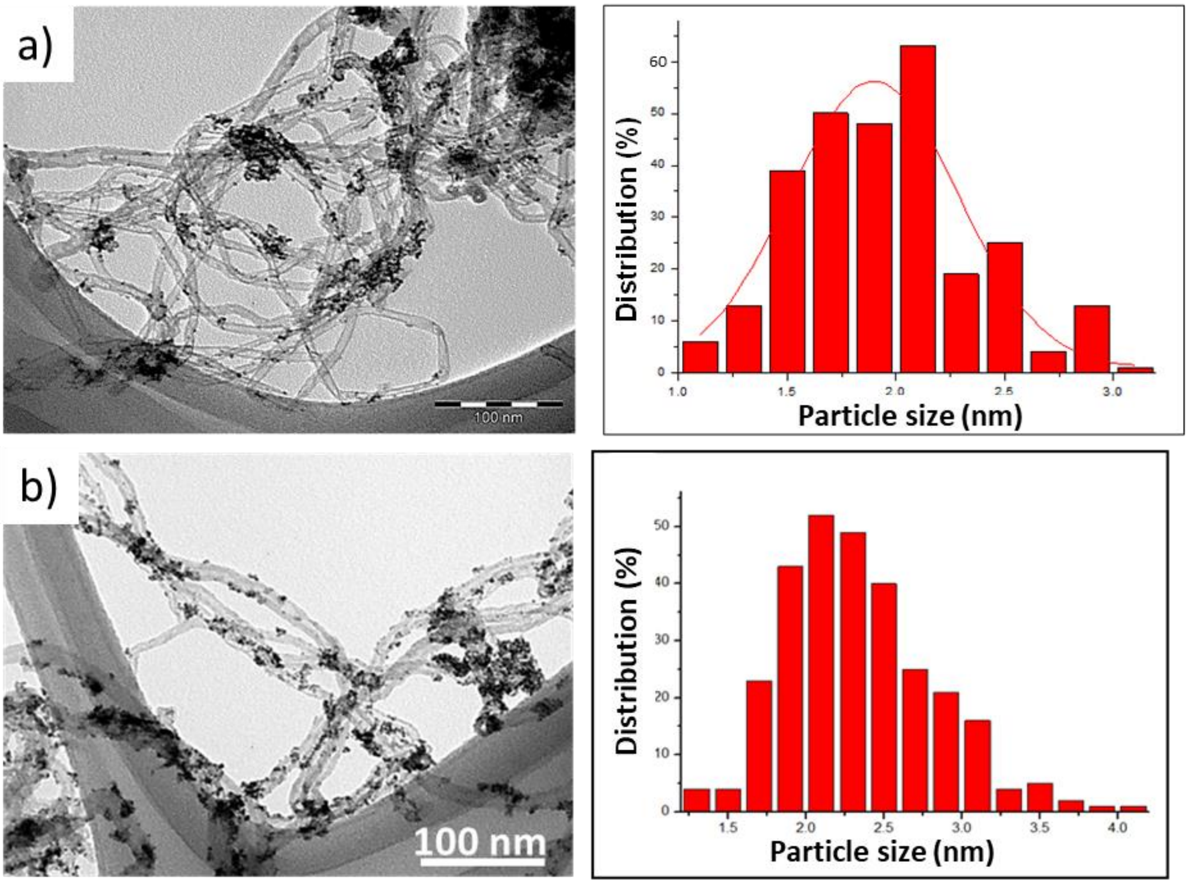

Figure S5: a) TEM images of Pt_3Co/CNT synthetized with hexadecyl trimetyl ammonium bromide as stabilizer, and size distribution for the catalyst (the mean particle size is 1.90 ± 0.77 nm). b) TEM images of Pt_3Co/CNT synthetized with [bmim][Tf_2N] as stabilizer, and size distribution for the catalyst (the mean particle size is 2.28 ± 0.82 nm).

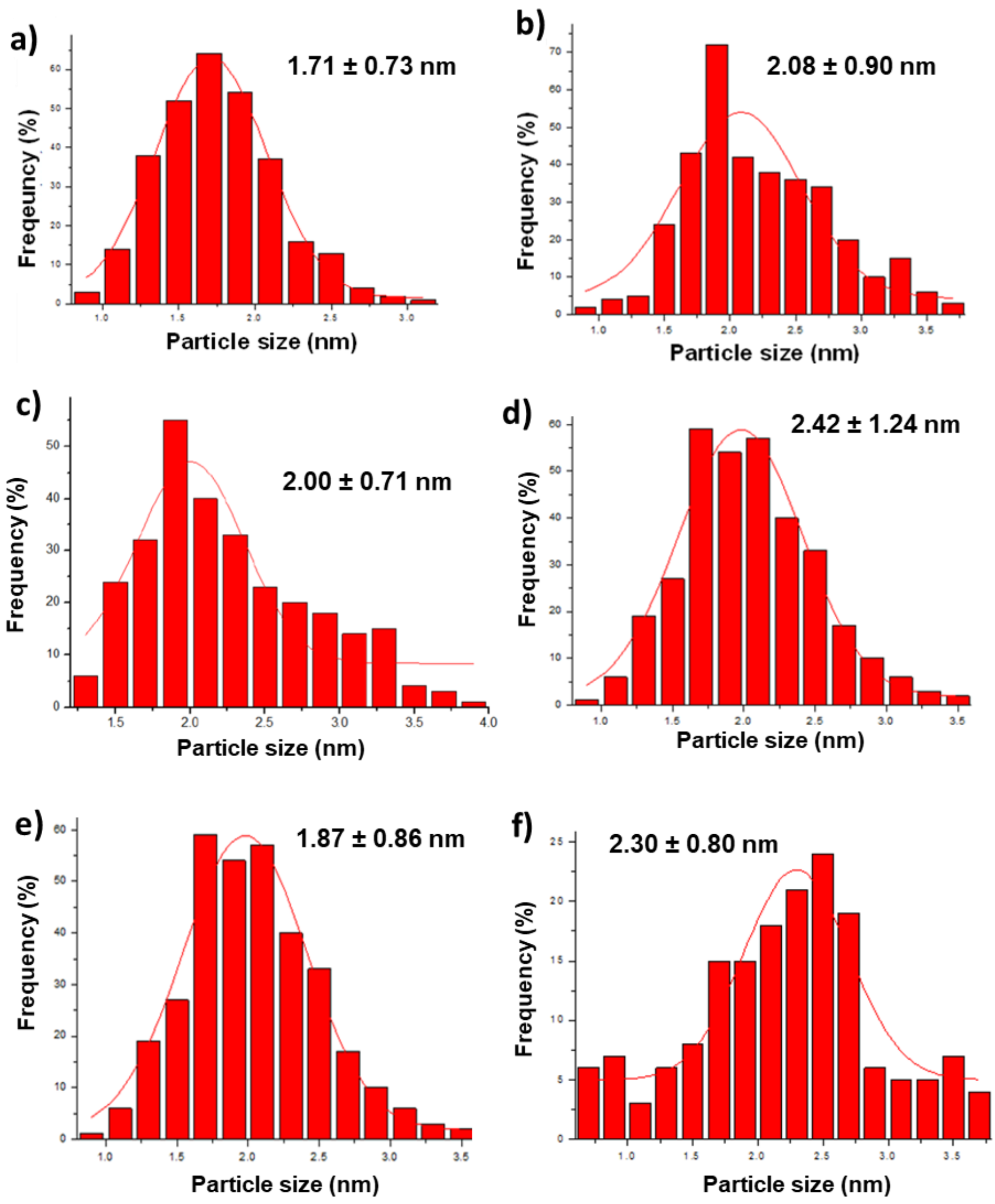

Figure S6: Size distribution for a) Pt_3Co/N-CNT, b) Pt_3Co/N-CNT_{HT}, c) Pt_3Co/S-CNT, d) Pt_3Ni/N-CNT, e) Pt_3Ni/N-CNT_{HT} and f) Pt_3Ni/S-CNT.

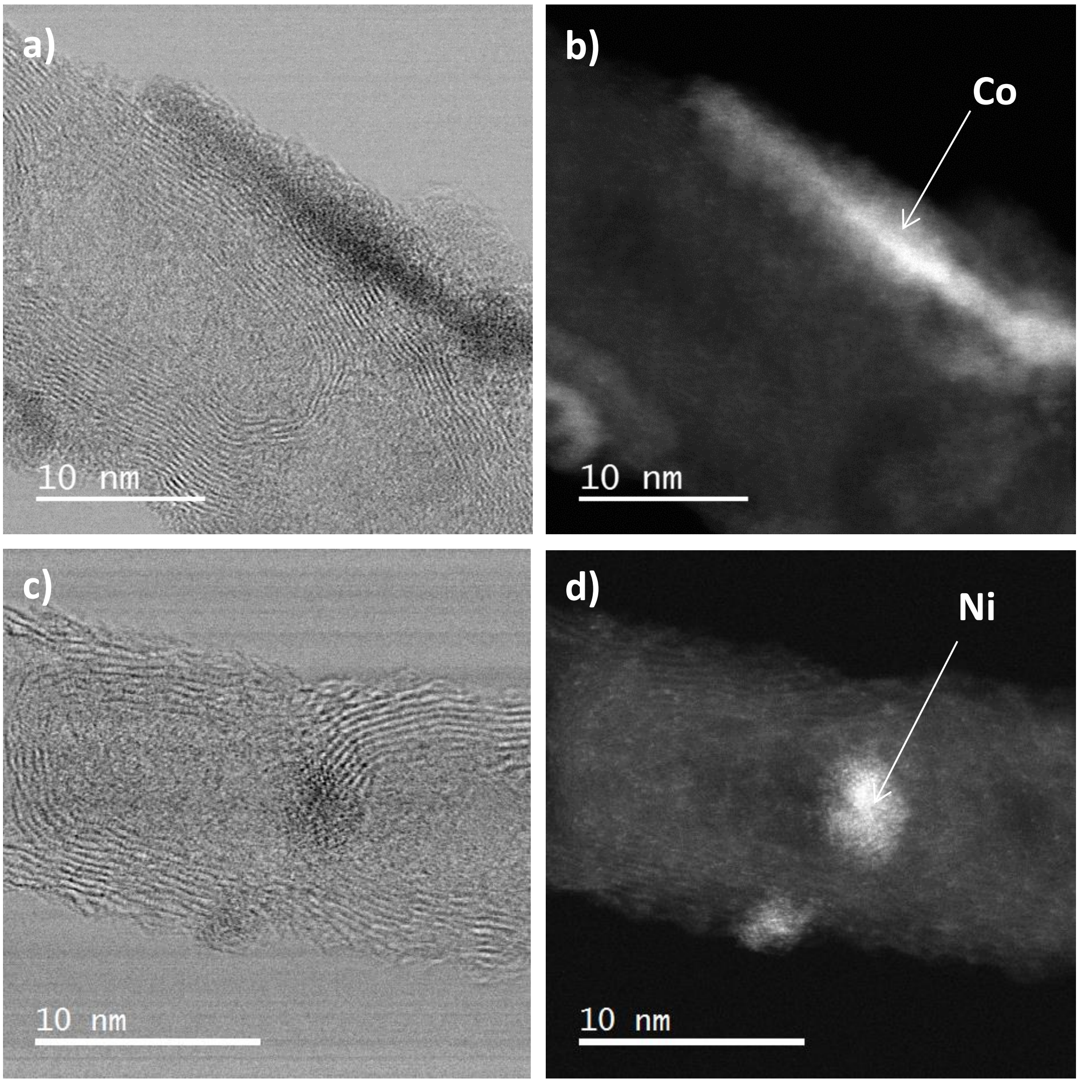

Figure S7: HRTEM images of a) Co/N-CNT and c) Ni/N-CNT$_{HT}$; and STEM-HAADF images of b) Co/N-CNT and d) Ni/N-CNT$_{HT}$.

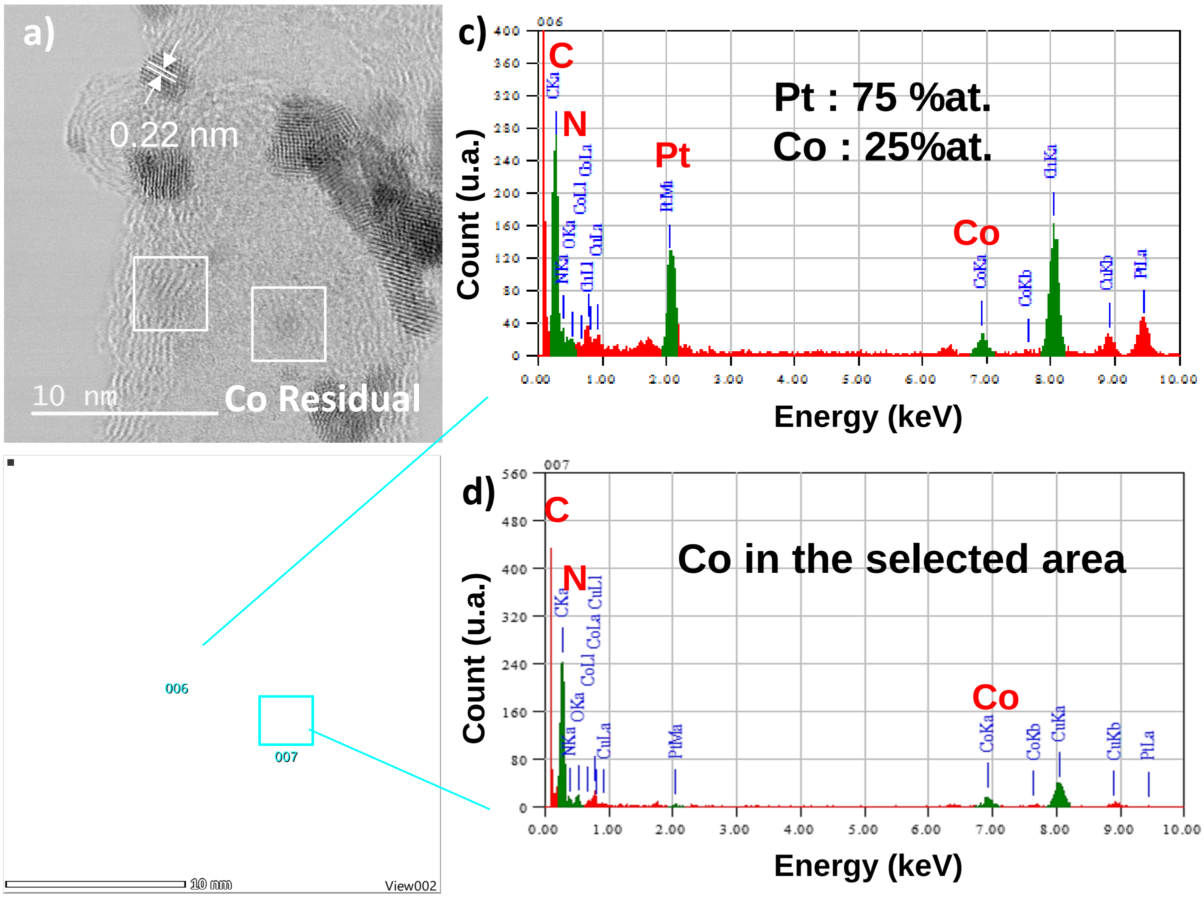

Figure S8: a) HREM image and b) STEM-HAADF micrograph of Pt_3Co/N-CNT and EDX spectrum of the selected area.

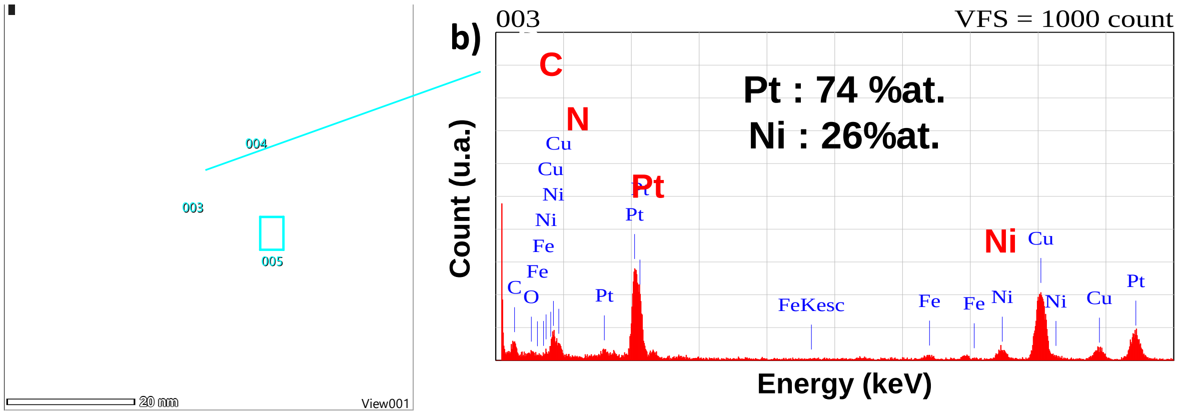

Figure S9: a) STEM-HAADF micrograph of Pt_3Co/N-CNT and b) EDX spectrum of the selected area.

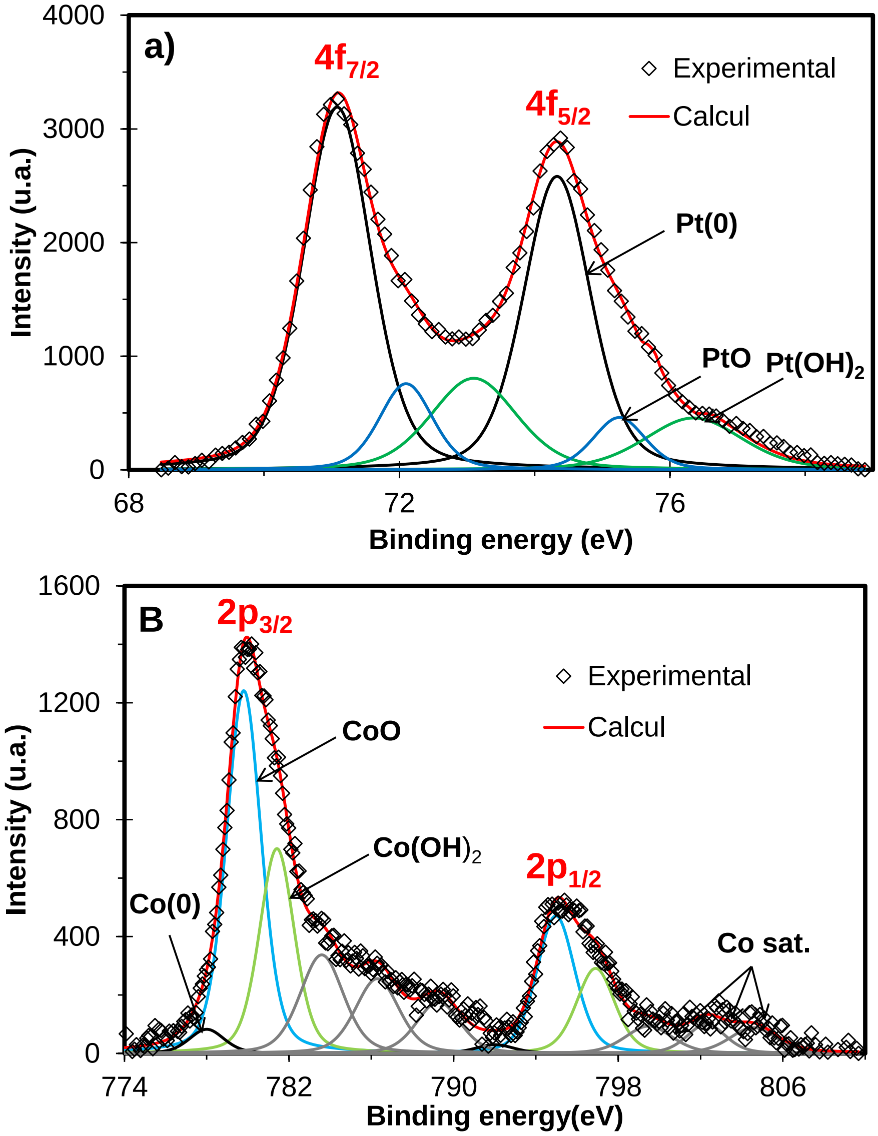

Figure S10: Deconvolution of XPS spectra for Pt_3Co/N-CNT a) Pt_{4f}, and b) Co_{2p}.

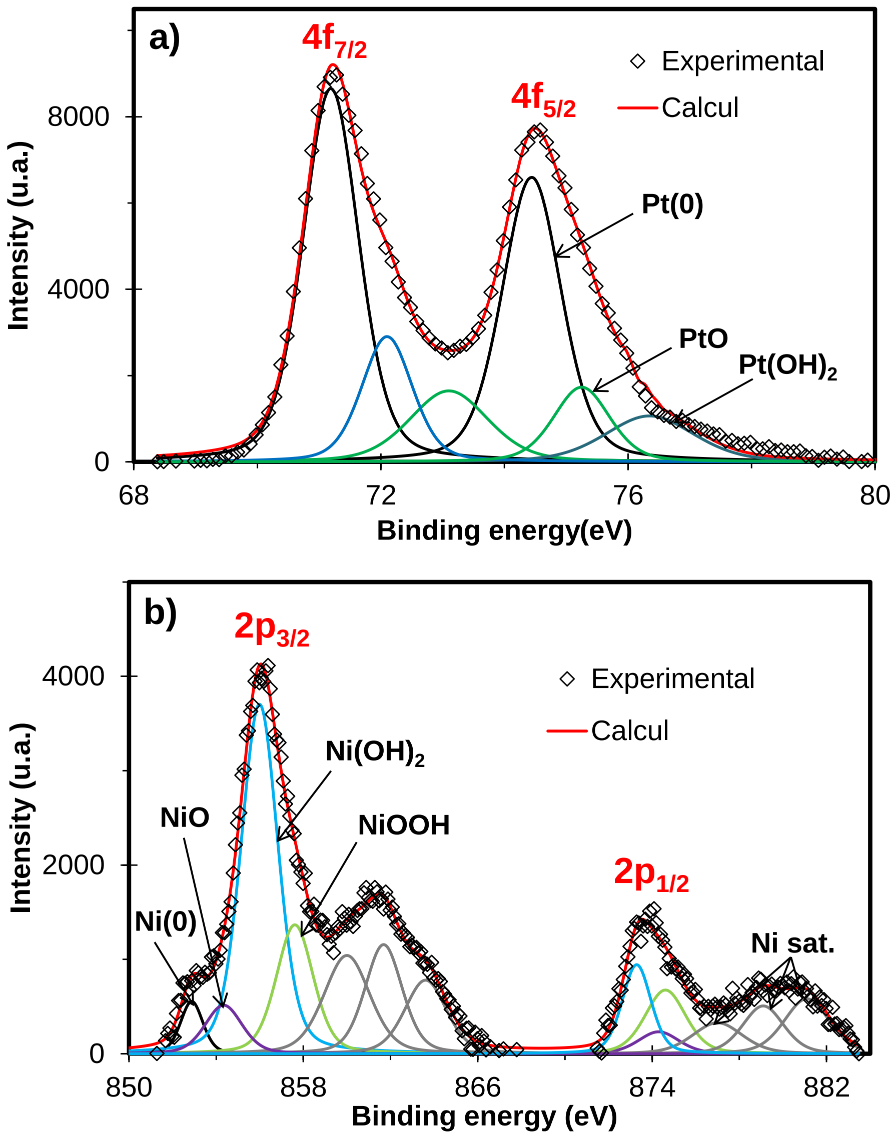

Figure S11: Deconvolution of XPS spectra for Pt_3Ni/N-CNT_{HT} a) Pt_{4f}, and b) Ni_{2p}.

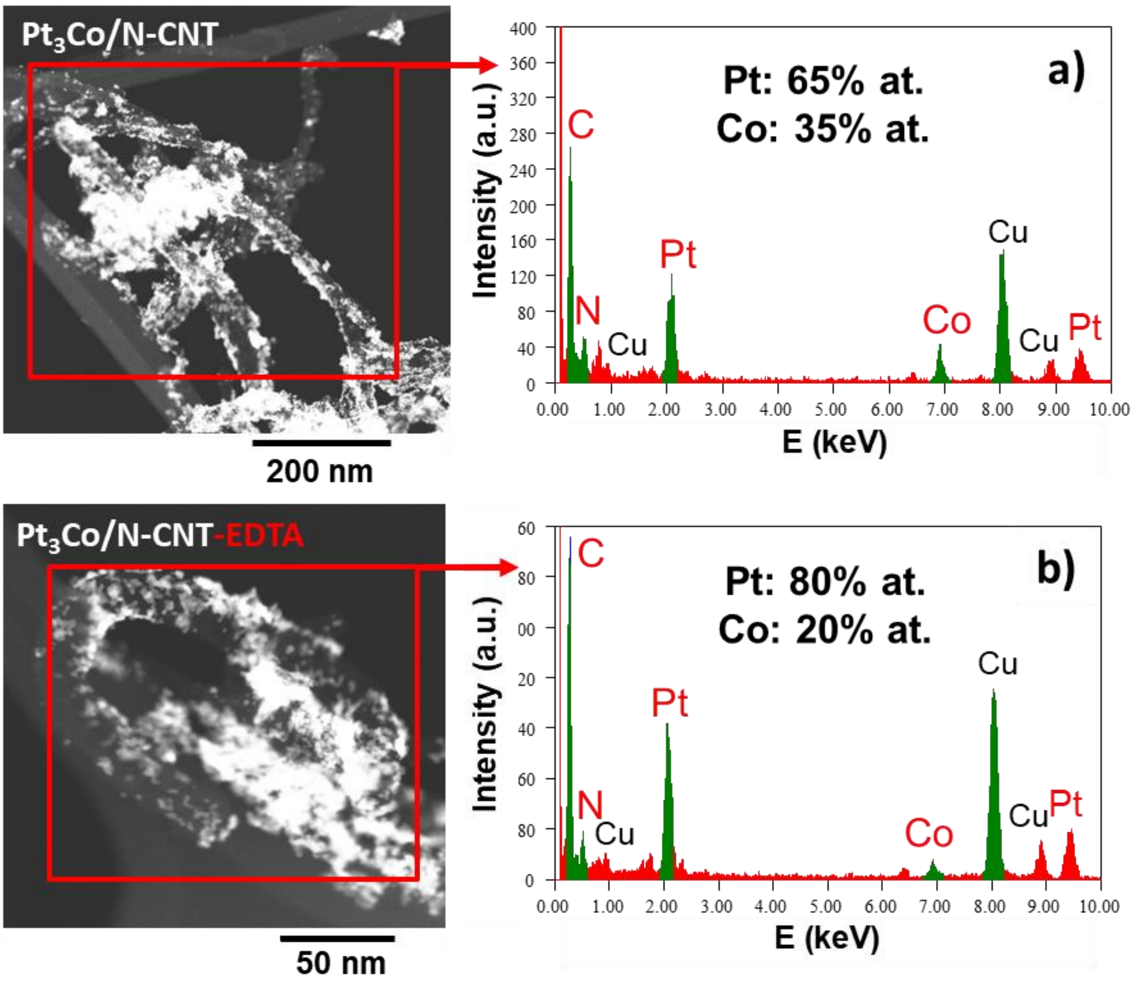

Figure S12: Results of the EDX analysis on Pt_3Co/N-CNT a) before and b) after washing with EDTA solution.

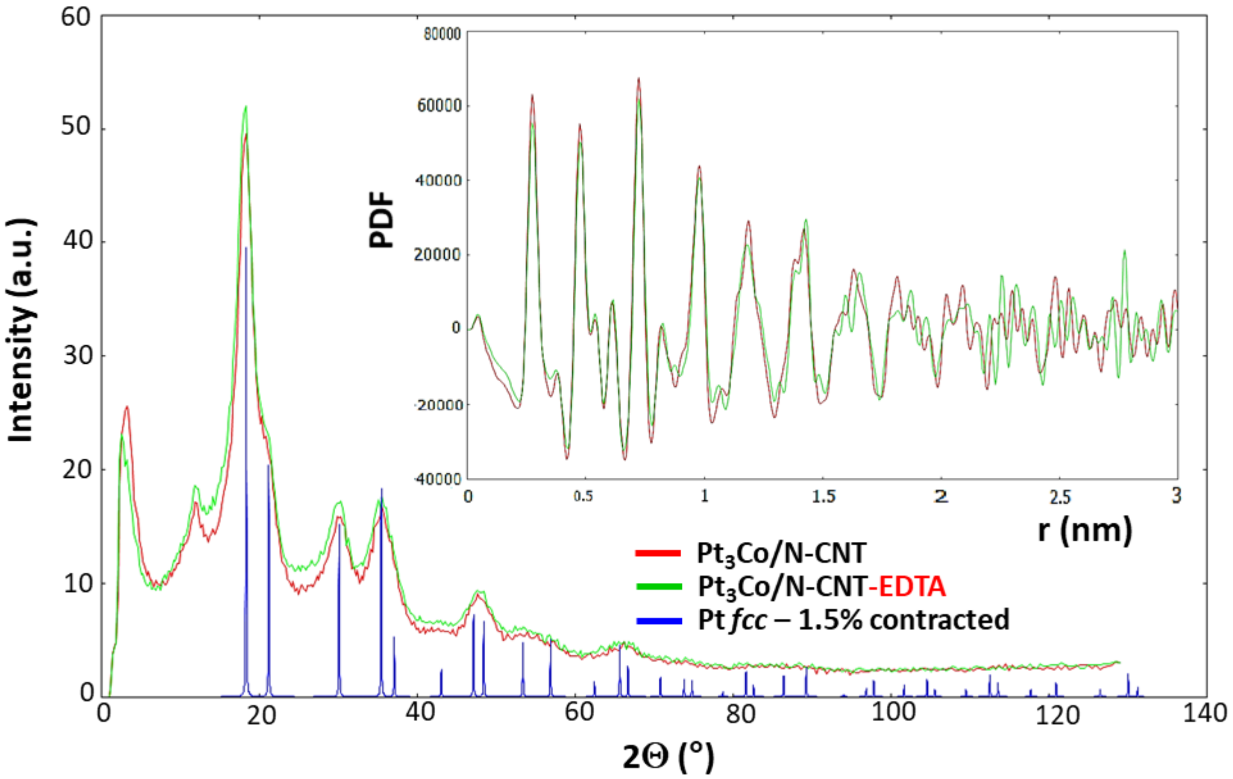

Figure S13: WAXS analyses – a) diffractogram from Pt_3Co/N-CNT before (red) and after (green) washing with EDTA and simulation for a spherical pure model; b) experimental PDF from Pt_3Co/N-CNT before (red) and after (green) washing with EDTA.

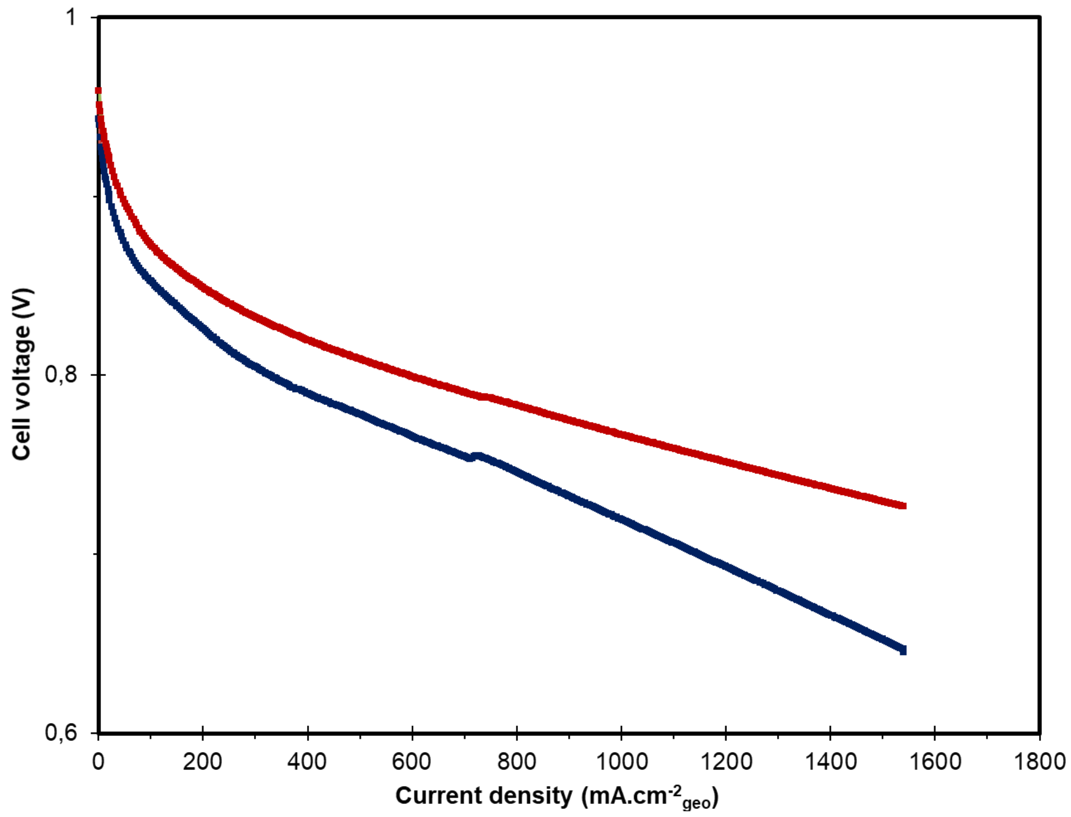

Figure S14: Polarization curves of MEA based on Pt_3Co/CB (red) and Pt_3Co/N-CNT (dark blue) ; recorded under O_2, P_{inlet} = 2.5 bar, T = 80 °C, St_{H2} = 1.2; St_{O2} = 5, RH_{anode} = 50%; $RH_{cathode}$ = 30%.

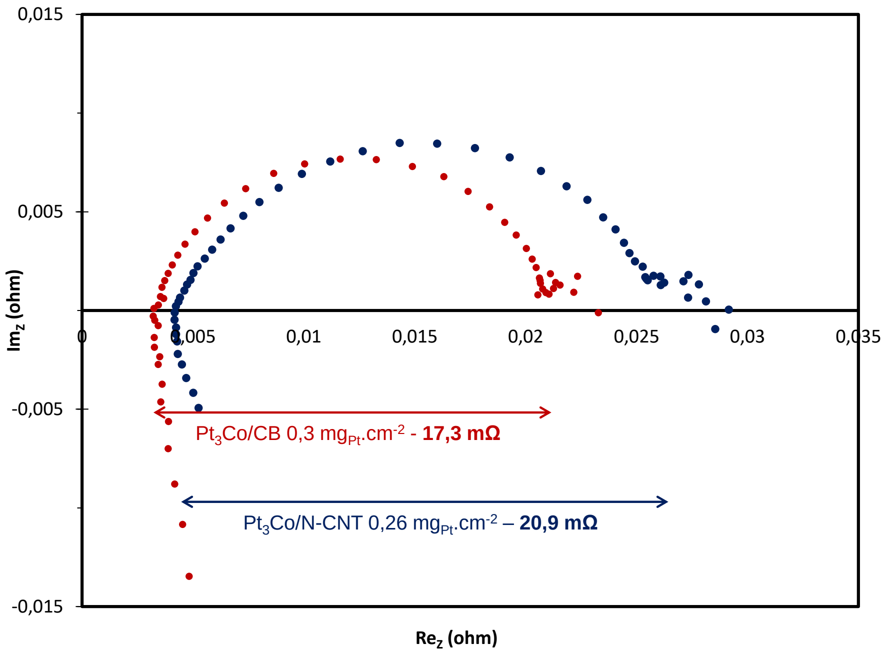

Figure S15: Nyquist diagrams of EIS for MEA based on Pt_3Co/CB (red) and Pt_3Co/N-CNT (dark blue) registered under air, P_{inlet} = 2.5 bar, T = 80 °C, St_{H2} = 1.2; St_{Air}= 3.5, RH_{anode} = 50%; $RH_{cathode}$ = 30%.
